# Virulence and Host Range of Fungi Associated With the Invasive Plant *Ageratina adenophora*

**DOI:** 10.3389/fmicb.2022.857796

**Published:** 2022-04-26

**Authors:** Lin Chen, Ai-Ling Yang, Yu-Xuan Li, Han-Bo Zhang

**Affiliations:** ^1^Key Laboratory of National Forestry and Grassland Administration on Biodiversity Conservation in Southwest China, Southwest Forestry University, Kunming, China; ^2^State Key Laboratory for Conservation and Utilization of Bio-Resources in Yunnan, Yunnan University, Kunming, China

**Keywords:** biocontrol agent, Didymellaceae, disease transmission, pathogen accumulation, plant–pathogen co-evolution

## Abstract

To determine whether disease-mediated invasion of exotic plants can occur and whether this increases the risk of disease transmission in local ecosystems, it is necessary to characterize the species composition and host range of pathogens accumulated in invasive plants. In this study, we found that Didymellaceae, a family containing economically important plant fungal pathogens, is commonly associated with the invasive plant *Ageratina adenophora*. Accordingly, we characterized its phylogenetic position through multi-locus phylogenetic analysis, as well as its environmental distribution, virulence, and host range. The results indicated that 213 fungal collections were from 11 genera in Didymellaceae, ten of which are known, and one is potentially new. *Didymella*, *Epicoccum*, *Remotididymella,* and *Mesophoma* were the dominant genera, accounting for 93% of total isolates. The virulence and host ranges of these fungi were related to their phylogenetic relationship. *Boeremia exigua, Epicoccum latusicollum,* and *E. sorghinum* were found to be strongly virulent toward all tested native plants as well as toward *A. adenophora*; *M. speciosa* and *M. ageratinae* were weakly virulent toward native plants but strongly virulent toward *A. adenophora*, thus displaying a narrow host range. Co-evolution analysis showed no strong phylogenetical signal between Didymellaceae and host plants. Isolates S188 and Y122 (belonging to *M. speciosa* and *M. ageratinae*, respectively) showed strong virulence toward *A. adenophora* relative to native plants, highlighting their potential as biocontrol agents for *A. adenophora* invasion. This study provides new insights into the understanding of the long-term ecological consequences of disease transmission driven by plant invasion.

## Introduction

Although the enemy release hypothesis, which postulates that invasive species have a competitive advantage in a new habitat because they leave their enemies from their native range behind, can explain some invasions (Keane and Crawley, 2002; Colautti et al., 2004), enemy accumulation after invasion is unavoidable (Mitchell et al., 2010; Flory and Clay, 2013; Stricker et al., 2016). This has led to increased interest in better understanding the pathogens accumulated by invasive plant species, as well as the pathogen-mediated ecological impacts.

Many invasive species can establish associations with native pathogens (Bufford et al., 2016; Dickie et al., 2017). Mitchell et al. (2010) analyzed fungal and viral pathogen species richness in 124 plant species in both native and introduced ranges and found that pathogen richness was correlated with host geographic range size, agricultural use, and time since introduction, but not with any of the measured biological traits. Regarding ecological effects, pathogen accumulation can have a direct role in plant invasion, including damaging the performance of the invasive host, such that invasion is initially slowed down, and then stopped, or even reversed (Hilker et al., 2005). This has been referred to as the “pathogen accumulation and invasive decline” hypothesis (Mitchell et al., 2010; Flory and Clay, 2013). For instance, pathogen infection of invasive *Microstegium* reportedly caused a significant decline in invasive host growth performance in natural populations (Flory et al., 2011). Additionally, Flory et al. (2018) demonstrated that emerging pathogens can suppress invaders and promote native species recovery.

Alternatively, pathogens can affect susceptible native hosts in the invaded ecosystem. Such dynamics are termed “spillover” when the pathogens are introduced with the invader and “spillback” when an invasive species hosts native pathogens and can further exacerbate the impact of the invasion (Flory and Clay, 2013). The general phenomenon has been called the “enemy of my enemy hypothesis” (Colautti et al., 2004; Flory and Clay, 2013).

Regardless of the roles of pathogen infection in plant invasion, both spillover and spillback processes suggest that invasive plants can serve as an ideal reservoir for native pathogens (Beckstead et al., 2010; Purse et al., 2013), which is likely to promote the emergence and amplification of novel pathogens in both agricultural and wild native plants. For example, Beckstead et al. (2010) reported that the invasive annual weed cheatgrass (*Bromus tectorum*) is a potential reservoir species for *Pyrenophora semeniperda*, a multiple-host, seed-borne fungal pathogen, which may have broad ecological consequences for plant community structure. Using maximum entropy models, Purse et al. (2013) showed that the invasive shrub *Rhododendron ponticum* can act as a host for the plant–pathogens *Phytophthora ramorum* and *P. kernoviae*, thereby increasing the vulnerability of affected ecosystems to disease spread.

The determination of the capacity of an invasive plant to act as a disease reservoir requires both a thorough understanding of the pathogen communities present in that invasive plant species, as well as the characterization of the host range of the pathogens that infect it. These efforts can help determine whether pathogen transmission is possible in local ecosystems and are also valuable for identifying potential biocontrol agents. However, relatively few studies have focused on determining the host ranges of pathogens. The few exceptions include Flory et al. (2011), who reported that *Bipolaris* species and other fungal pathogens isolated from invasive plants of the genus *Microstegium* could cause disease in native grasses and forbs. The authors reported that although *Bipolaris* species could be effective as a pathogen against *Microstegium*, its potential use as a biocontrol agent was limited because of its effects on a wide range of grasses, including corn, sorghum, rye, and wheat (Kleczewski et al., 2012).

*Ageratina adenophora* Spreng (family Asteraceae) (basionym: *Eupatorium adenophorum* Spreng) is one of the most invasive weeds in China (Wang and Wang, 2006). It is estimated to cause economic losses to animal husbandry and grassland ecosystem services of RMB 0.99 and 2.63 billion per year, respectively (Wang et al., 2017). *Ageratina adenophora* has been reported to host the leaf spot-causing pathogenic fungi *Alternaria alternata* (Wan et al., 2001; Zhou et al., 2010) and *Passalora ageratinae* (Buccellato et al., 2021). Members of the genus *Didymella* were also shown to inhibit seedling growth in *A. adenophora* (Fang et al., 2019). Our recent investigation indicated that *A. adenophora* hosts a wide variety of fungal pathogens, particularly those belonging to the family Didymellaceae, found to be abundantly present in leaf spots and the healthy leaves of *A. adenophora* (Chen et al., 2020). The Didymellaceae comprise the largest family within the order Pleosporales (Ascomycota, Pezizomycotina, and Dothideomycetes), with more than 3,000 names listed in MycoBank, in only one genus, *Phoma* (Valenzuela-Lopez et al., 2018). Didymellaceae species are distributed throughout a broad range of environments and most are economically important plant–pathogenic fungi with a wide host range, mainly causing leaf and stem lesions (Chen et al., 2015, 2017). However, the host range of Didymellaceae fungi isolated from *A. adenophora* remains unclear.

We have previously demonstrated that fungi associated with *A. adenophora* are also widely present in native plants, thus representing an increased risk of disease to the latter (Chen et al., 2020). Meanwhile, a numerically dominant but unclassified OTU (OTU515) belonging to the family Didymellaceae was repeatedly isolated from *A. adenophora* but not from native plants, suggesting that it may be host-specific (Chen et al., 2020). These observations further implied that these fungi could be developed as biocontrol agents targeting *A. adenophora* if they are indeed host-specific; alternatively, if they are instead generalists, they represent a high disease risk in local ecosystems. However, all Didymellaceae species have been defined based on the sequencing of the internal transcribed spacer (ITS) locus, and the *rpb2* gene has since been reported to be a more effective locus for identifying Didymellaceae fungi at both the intra- and interspecific levels (Hou et al., 2020). Moreover, whether all these fungi from one genus display narrow phylogenetic distribution or high phylogenetic diversity remains unknown, rendering it difficult to link host range and virulence with phylogeny. Accordingly, in this study, we aimed to (1) characterize the phylogenetic status of the Didymellaceae isolates associated with *A. adenophora*, as well as those obtained from native plants and the local environment, including soil and air, using multiple loci; (2) link the virulence and host range of the isolates with the relevant phylogeny. Our work greatly improves the understanding of pathogen accumulation in *A. adenophora* and the disease-associated ecological risks, and greatly contributes to the identification of potential fungal biocontrol agents targeting *A. adenophora*.

## Materials and Methods

### Sampling and Isolation

Fungal strains were isolated from 18 sampling sites in Yunnan province, China, from 2012 to 2017 (Supplementary Table 1). Isolates were obtained from healthy leaves, leaf spots, the stem, and leaf litter of *Ageratina adenophora*. The canopy air and rhizosphere soil associated with *A. adenophora* were sampled, as were the leaf spots of native plants from invaded and noninvaded ranges. Native plants growing immediately next to *A. adenophora* were selected for leaf spot collection. If such plants were unavailable, native plants growing close (<5 m) to *A. adenophora* were selected instead.

To isolate leaf-based endophytes, the fourth pair of healthy leaves from the top of fully grown *A. adenophora,* which harbor the greatest fungal diversity (Jiang et al., 2011), were collected from July to August. Endophytes were obtained using the leaf fragment incubation technique (Arnold and Lutzoni, 2007). To obtain sufficient pathogen diversity, in any one sample site, diseased leaves with morphologically different symptoms were collected from at least three individuals. Healthy leaf tissues and the margins of diseased tissues of each leaf spot were cut into six sections (6 mm^2^) and the surface was sterilized. The sterile fragments were then plated into PDA and incubated at ambient temperature for 6–8 days or until mycelia were observed growing from the leaf fragments.

For collection of the rhizosphere soil, plants were dug out and lightly shaken, and the soil that remained attached to the root surface was carefully collected using a brush. Rhizosphere soils were packed into polyethylene bags and placed on ice packs in a cooler after collection and immediately transported to the laboratory where they were stored at 4°C until processing. To obtain soil suspensions, soil samples (10 g) were placed in triangular bottles containing 100 ml of sterile water and several glass beads and incubated at 28°C for 30 min with shaking (180 rpm). Then, 0.1 ml of soil suspension (10^−2^ and 10^−3^ dilutions) was placed on a PDA dish and incubated at ambient temperature for 6–8 days. Fungi were collected from the air by exposing PDA Petri dishes 60 cm above the canopy for 15 min. The Petri dishes were then sealed using a sealing membrane and returned to the laboratory where they were incubated at 28°C for 3–4 days.

All fungal colonies were purified and used for the determination of their phylogenetic position based on the ITS locus sequence. Those belonging to the family Didymellaceae were selected and further subjected to multi-locus phylogenetic analyses and disease experiments. All fungi were maintained as pure cultures at Yunnan University (Kunming, China).

### DNA Isolation, Amplification, and Multi-Locus Phylogenetic Analysis

Total genomic DNA was extracted from fresh mycelia using the CTAB method (Murray and Thompson, 1980). The first ITS (ITS1) region was amplified with the ITS4 and ITS5 primers (White et al., 1990); LR5 and LR7 (Vilgalys and Hester, 1990) were used for large subunit (LSU) rRNA (LSU rRNA) amplification; Btub2Fd and Btub4Rd (Woudenberg et al., 2009) were used for the partial amplification of the β-tubulin (*tub2*) gene; and finally, RPB2-5F2 (Sung et al., 2007) and fRPB2-7cR (Liu et al., 1999) were used for the amplification of the second-largest subunit of RNA polymerase II (*rpb2*). Amplicons for each locus were generated by Sanger sequencing following the protocols listed in Chen et al. (2017). Before multi-locus phylogenetic analyses, the sequences of all the loci generated in this study were used as queries to search similar DNA sequences in GenBank using BLAST. The isolates displaying the highest sequence similarity to Didymellaceae species were selected for analysis. The sequences of isolates and reference strains based on Chen et al. (2017) and Valenzuela-Lopez et al. (2018) were aligned and combined using BioEdit version 7.0 (Hall, 1999).

Bayesian (BI) analysis was performed on MrBayes v.3.2.1 (Ronquist et al., 2012) based on the models selected by MrModeltest v.2.3 according to the protocol described by Chen et al. (2017). The best-fit models of evolution for the four loci tested (GTR + I + G for ITS, LSU, *rpb2*, and *tub2*) were estimated by MrModeltest. *Leptosphaeria conoidea* (CBS 616.75) and *L. doliolum* (CBS 505.75) were selected as outgroup species. To identify the phylogeny of the isolates, the fungi identified by Chen et al. (2017) were used as the reference strains in the phylogenetic analysis (Supplementary Table 2).

### Evaluating the Virulence of Didymellaceae Against *Ageratina adenophora* and Native Plants

Based on the multi-locus phylogenetic analysis, 131 isolates belonging to 11 genera were selected to evaluate virulence against *A. adenophora* and 14 native plant species commonly found in Yunnan province (Institute of Botany, 2019), including three trees [*Celtis tetrandra* (Ulmaceae), *Cyclobalanopsis glauca* (Fagaceae), and *Lindera communis* (Lauraceae)], five vines [*Ampelopsis delavayana* (Vitaceae), *Fallopia multiflora* (Polygonaceae), *Petunia hybrid* (Solanaceae), *Pueraria peduncularis* (Fabaceae), and *Zehneria maysorensis* (Cucurbitaceae)], five shrubs [*Urena lobata* (Malvaceae), *Abelmoschus moschatus* (Malvaceae), *Achyranthes bidentata* (Amaranthaceae), *Rubia cordifolia* (Rubia cordifolia), and *Reinwardtia indica* (Linaceae)], and one grass [*Arthraxon hispidus* (Poaceae)]. A disease experiment was performed in the field as previously reported for the testing of the virulence of necrotrophic pathogens in tropical forests (Gilbert and Webb, 2007), with a minor modification. Briefly, fungi were grown on PDA for 7 days, after which agar disks (6 mm^2^) containing fungal mycelia were used to inoculate mature and healthy leaves. Small wounds were made by lightly touching the underside of the leaf with a sterilized toothpick, resulting in a wound area of ~0.2 cm^2^. The inoculum agar was pressed against the wound on the underside of the leaf using Scotch tape and clipped in place with a bent hair clip. Control inoculation was performed with agar without fungal mycelia. One week after inoculation, the leaves were harvested and photographed, and symptomatic areas were measured. Because the control inoculation showed only a minimal wound reaction, strains inducing significant wound symptoms were considered pathogenic. The field site was located in Xishan Forest Park, Kunming, at an altitude of 2,214 m, a latitude of 24°58′24″ N, and a longitude of 102°37′17″ E. The experimental period ran from June to the end of October 2017–2018, the primary growing season for plants in Kunming.

After this analysis, 13 potential biocontrol agents were screened for virulence in an additional 15 hosts that included *Alnus nepalensis* (Fagales), *Amphicarpaea edgeworthii* (Fabaceae), *Bambusa emeiensis* (Poaceae), *Cynanchum otophyllum* (Apocynaceae), *Hypoestes triflora* (Acanthaceae), *Michelia figo* (Magnoliaceae), *Myrica rubra* (Myricaceae), *Oreocnide frutescens* (Urticaceae), *Parthenocissus tricuspidata* (Vitaceae), *Quercus variabilis* (Fagaceae), *Rosa xanthina* (Rosaceae), *Rubus parvifolius* (Rosaceae), *Sechium edule* (Cucurbitaceae), *Smilax scobinicaulis* (Smilacaceae), and *Zanthoxylum bungeanum* (Rutaceae). The disease experiment was performed as described above. Four *Epicoccum* isolates and one *Didymella* isolate served as positive and negative controls, respectively.

To screen candidates for their biological control potential against *A. adenophora*, three isolates with high virulence and a narrow host range were selected for the validation of virulence *in vitro* in leaves of *A. adenophora*. Meanwhile, to determine if the host, *A. adenophora*, evolves resistance to fungal virulence, the tested plants were grown from seeds collected from *A. adenophora* populations with 40 and 80 years of invasion history (Wang and Wang, 2006). Fresh hyphae (3 g) were crushed in a magnetic stirrer and diluted in 12 ml of a solution containing 10% sterilized glycerinum. A volume of 10 μl of glycerinum with hyphae was dripped into the back of injured and uninjured leaves that had been placed in a Petri dish moistened with 10% glycerin and sterilized filter paper. The size of the diseased spot was recorded daily. Three or more replicates were set for each strain. Because leaf spots covered whole leaves in some isolates by day 5 after inoculation, day 4 was selected for the evaluation of virulence against *A. adenophora* of differing invasion histories. Meanwhile, 10 ml of glycerinum with hyphae was used to spray 12 plants (6 with injured and 6 with uninjured leaves) and the disease spots were recorded. All leaf spots were reisolated, thus achieving Koch’s postulates.

### Data and Statistical Analysis

To construct a plant–pathogen evolutionary association network, three DNA sequences—*rbcL*, *matK*, and *ITS1*—were obtained from GenBank for each of the 15 plant species (Supplementary Table 2). Two early-diverging gymnosperm species—*Abies alba* and *Cycas rumphii—*served as the outgroup (Chen et al., 2019). Also, isolates used for the evaluation of virulence were selected for further analysis. The phylogenetic tree of the hosts was then built using the RAxMLGUI v1.5 algorithm (Stamatakis, 2014) under a maximum likelihood model with a General Time Reversible (GTR) + Gamma nucleotide substitution model of evolution (1,000 bootstrap replicates). After removing the two outgroup species, a final molecular phylogeny was obtained for the 15 host species. The phylogenetic tree of all these pathogenic isolates was built using BI analysis, as mentioned above. Global and individual ParaFit tests (Legendre et al., 2002; Chen et al., 2019) were performed to examine whether there was a non-random plant–pathogen association network.

Heatmaps for leaf spot sizes were plotted using the R package pheatmap (Kolde, 2018). The Kruskal-Wallis test (for three and more groups) and the Mann–Whitney U test (for two groups) were used to test between-group differences in SPSS v.25.0 (IBM, Chicago, IL, USA). Diagrams were plotted in GraphPad Prism 7 (GraphPad Software, Inc., La Jolla, CA, USA).

## Results

### Fungal Isolation and Multi-Locus Phylogenetic Analysis

A total of 213 Didymellaceae isolates were collected, including 189 (89%) from 12 sites with and 24 (11%) from 6 sites without *Ageratina adenophora* invasion. Of these, 195 (92%) were obtained from plant samples and 18 (8%) from environmental samples, including rhizosphere soil (2 isolates) and air (16 isolates). Most of the strains (145 isolates) were isolated from *A. adenophora* (101 from leaf spots, 29 from healthy leaves, 5 from stems, 7 from roots, and 3 from rotted leaves); 50 isolates were obtained from native plant leaf spots, including 26 from invaded ranges and 24 from noninvaded ranges (Supplementary Tables 3 and 4).

**Figure 1:**
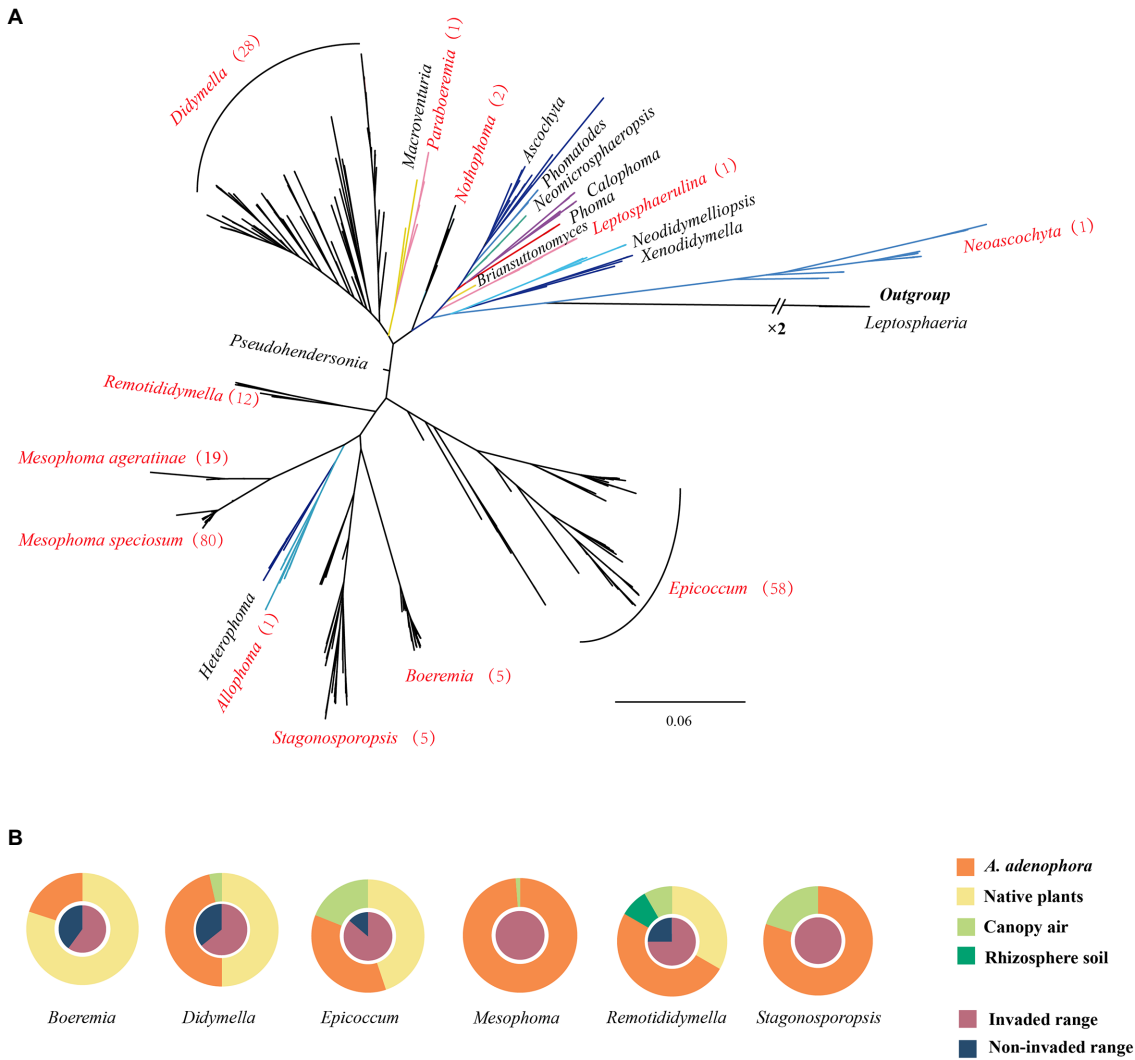
Phylogenetic tree and source of the Didymellaceae isolates. **(A)** Phylogenetic tree of Didymellaceae. Genera names in red represent the distribution of the isolates collected in this study and the numbers in brackets represent the number of strains isolated. For additional details, please see Supplementary Figures 1, 2. **(B)** Source of the Didymellaceae isolates.

Phylogenetically, as defined by ITS sequencing, a total of 52 unique OTUs and 6 OTUs based on 97% similarity were obtained. Multi-locus analysis identified a total of 131 OTUs (Supplementary Table 5). These fungi were distributed in 10 genera, including *Epicoccum* (58 isolates, 27%), *Didymella* (28 isolates, 13%), *Remotididymella* (12 isolates, 6%), *Boeremia* (5 isolates, 2%), *Stagonosporopsis* (5 isolates, 2%), *Nothophoma* (2 isolates, 0.5%), *Allophoma* (1 isolate, 0.5%), *Leptosphaerulina* (1 isolate, 0.5%), *Neoascochyta* (1 isolate, 0.5%), and *Paraboeremia* (1 isolate, 0.5%), as well as two new groups (named *Mesophoma speciosa*, 80 isolates, 37%; *M. ageratinae*, 19 isolates, 9%) (Supplementary Figures 1, 2, the novel genus report in other article Yang et al., 2022 pers. comm.). Isolates belonging to *Didymella*, *Epicoccum*, *Remotididymella*, and *Mesophoma* accounted for 93% of the total isolates (Figure 1A and Supplementary Table 5. The distribution of the seven main groups is shown in Figure 1B. *Epicoccum*, *Didymella*, *Remotididymella*, and *Boeremia* were isolated from both invaded and noninvaded ranges, as well as from leaf spots of both *A. adenophora* and native plants. However, isolates belonging to *Stagonosporopsis*, *M. speciosa*, and *M. ageratinae* were only collected from sites with *A. adenophora* invasion. *Epicoccum*, *Didymella*, and *Stagonosporopsis* isolates were obtained from canopy air, while *Remotididymella* was found in rhizosphere soil and canopy air.

### Evaluating the Virulence of Didymellaceae Against *Ageratina adenophora* and Native Plants

**Figure 2:**
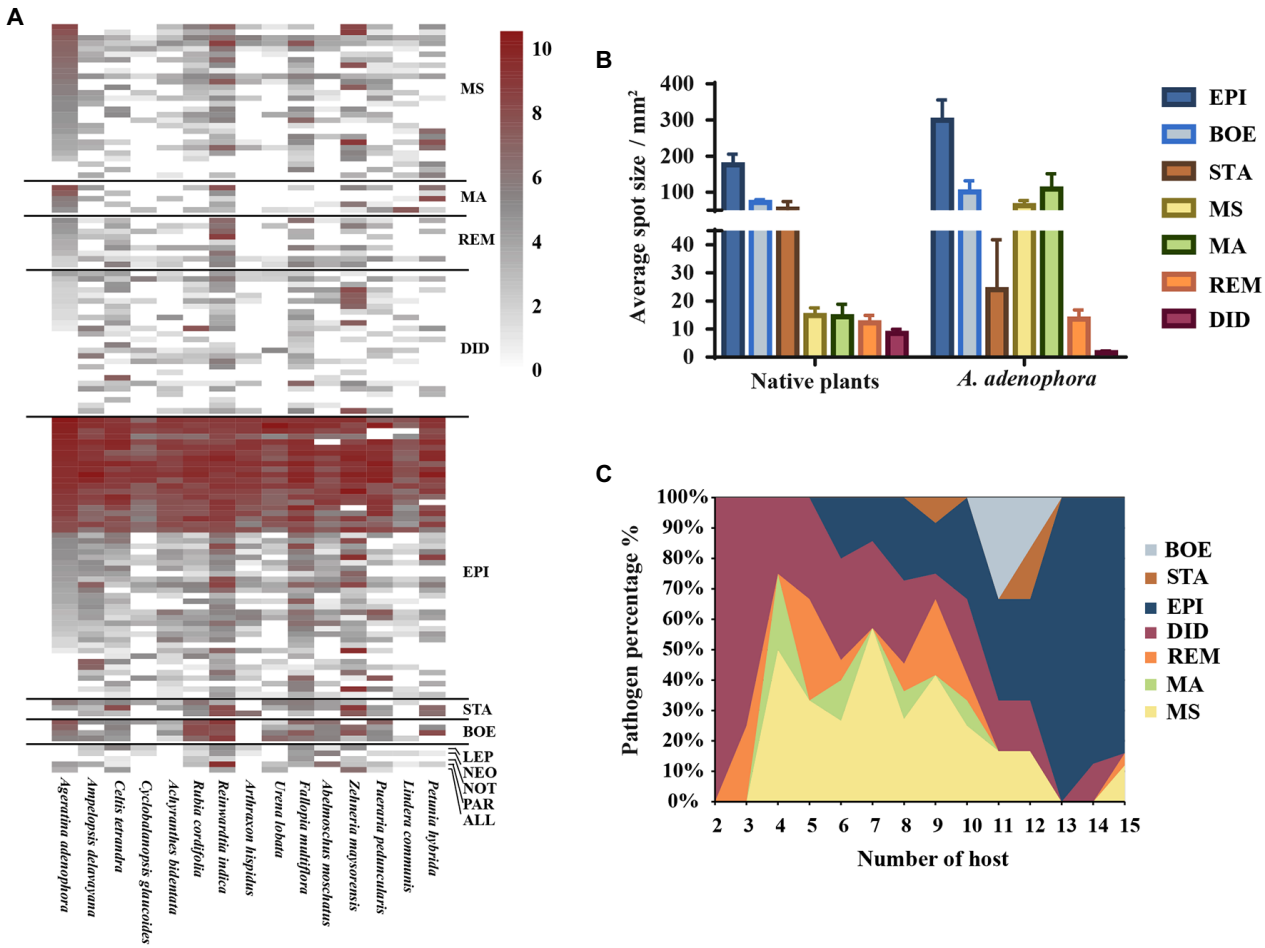
Evaluating the virulence of 131 Didymellaceae toward *Ageratina adenophora* and 14 native plants. **(A)** Heatmap of leaf spot sizes transformed by log_2_(x + 1). **(B)** Average spot size of genera (≥3 isolates) on native plants and *A. adenophora*. **(C)** Percent pathogen distribution in the 15 different hosts. ALL, *Allophoma*; BOE, *Boeremia*; DID, *Didymella*; EPI, *Epicoccum*; LEP, *Leptosphaerulina*; MA, *Mesophoma ageratinae*; MS, *M. speciosa*; NEO, *Neoascochyta*; NOT, *Nothophoma*; PAR, *Paraboeremia*; REM, *Remotididymella*; and STA, *Stagonosporopsis.* Error bars represent 1 SE.

**Figure 3:**
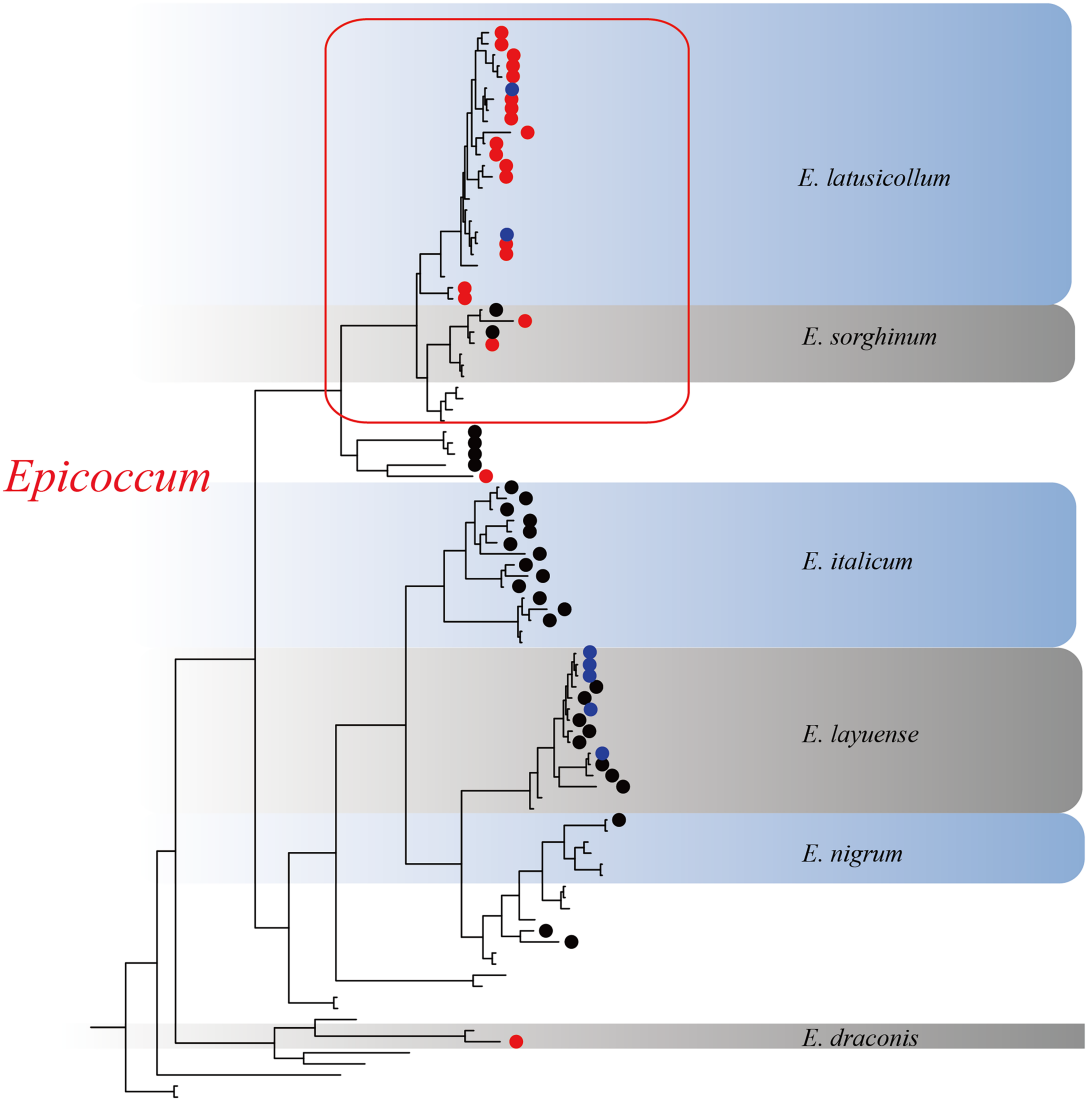
The *Epicoccum* clade in the phylogenetic tree. Red circles represent strongly virulent isolates, black circles represent weakly virulent isolates, blue circles represent isolates for which virulence was not evaluated in this study, and nodes without symbols represent the standard sequences downloaded from GenBank.

Based on the multi-locus phylogenetic analysis, 131 typical isolates from both known genera and unclassified groups were selected for the field disease experiment (Figure 2). These fungi varied greatly in virulence and host range, even those in the same genus (Figure 2A). In particular, 21 strains belonging to *Epicoccum* showed extremely strong virulence and broad host range, and clustered primarily in a small clade phylogenetically close to species *E. latusicollum* and *E. sorghinum* (Figure 3, red frame).

**Figure 4:**
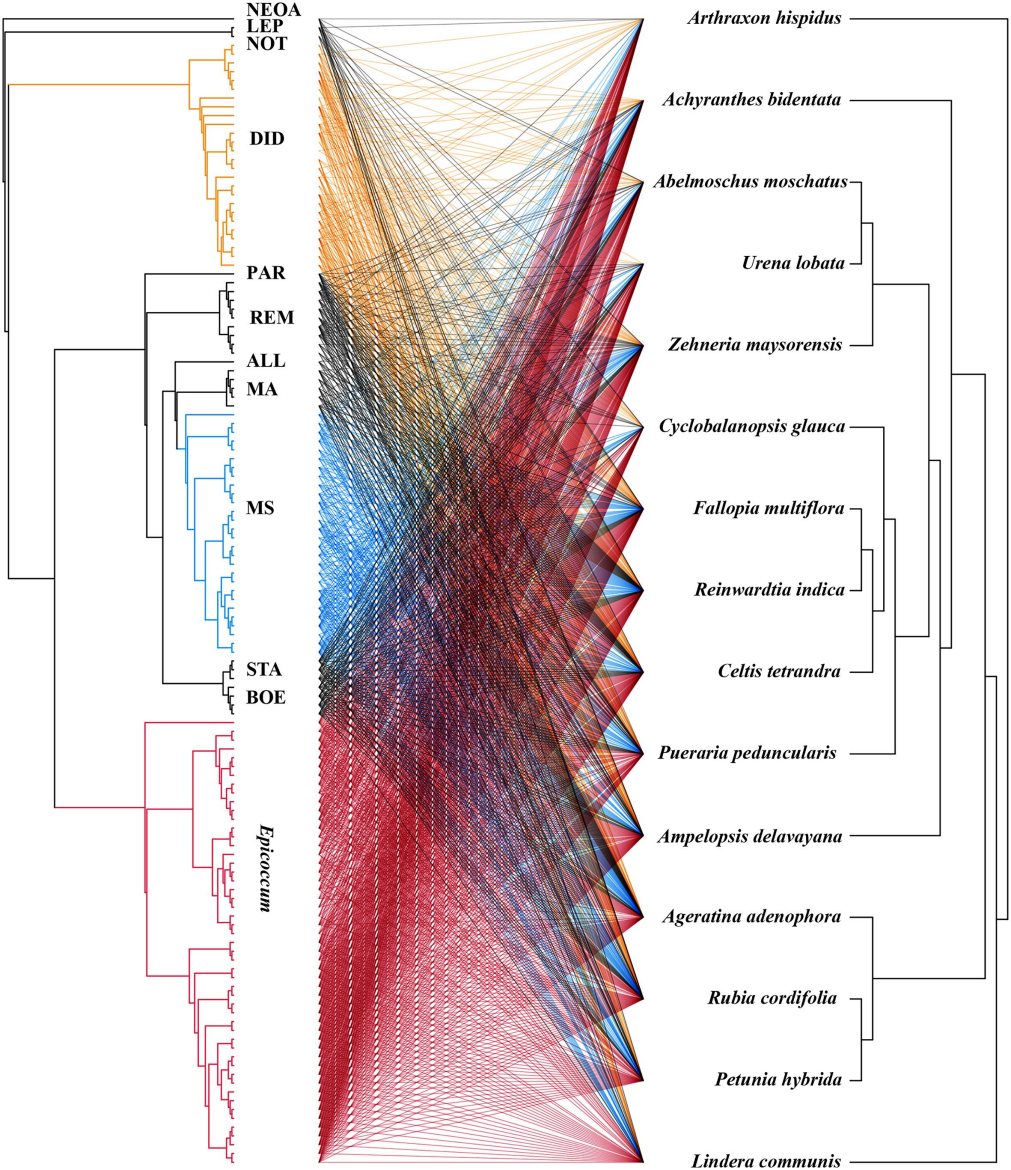
Co-evolution analysis of Didymellaceae pathogens and host plants. The tree on the right shows the fungal phylogenetics and the tree on the left shows the plant phylogenetics. The lines in the middle represent the pathogenic relationships between fungi and host plants.

The virulence of fungi against *A. adenophora* and native plants differed significantly among genera (*p* = 0.000, Kruskal-Wallis test) (Figure 2B). Both *Boeremia* and *Epicoccum* were strongly virulent toward all tested native plants, including *A. adenophora*; in contrast, *Remotididymella* and *Didymella* were only weakly virulent toward all tested hosts. Interestingly, most members from *M. speciosa* and *M. ageratinae* were weakly virulent toward native plants but strongly virulent against *A. adenophora*. The genus *Stagonosporopsis* was associated with median spot size in *A. adenophora* and large spot size in native plants. Host range also differed significantly among genera (*p* = 0.000, Kruskal-Wallis test; Figure 2C). Genera exhibiting relatively high virulence, namely, *Boeremia*, *Epicoccum*, and *Stagonosporopsis*, showed a wider host range than those displaying weak virulence (*Didymella* and *Remotididymella*); as the host number increased, *Epicoccum* replaced *Didymella* as the dominant pathogenic fungus. *M. speciosa* and *M. ageratinae* had a median host range (Figure 2C). There was no strong phylogenetic correlation between pathogen and host (global ParaFit tests, *p* = 1), i.e., a wide range of phylogenetic strains could infect the same host, and the same phylogenetic strain could infect a wide range of hosts (Figure 4). Relatively, *R. indica* was the most susceptible host and 92% of the isolates were virulent against it; in contrast, *C. glauca*, with only 44% of virulent isolates, was the most resistant to Didymellaceae infection.

**Figure 5:**
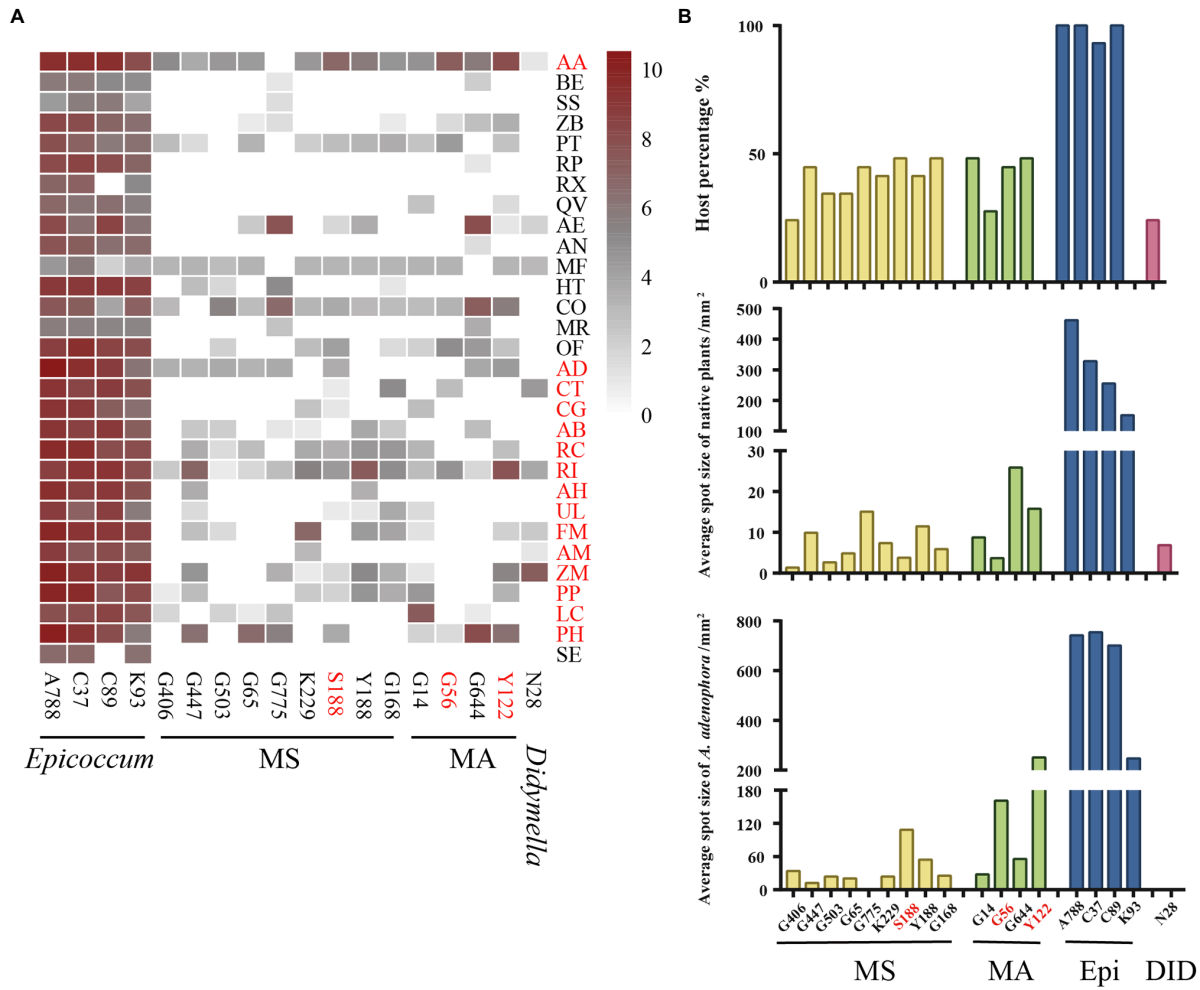
Evaluating the virulence of 18 Didymellaceae against *Ageratina adenophora* and 29 native plants. **(A)** Heatmap of leaf spot sizes transformed by log_2_(x + 1). **(B)** Host percentage and average spot size of typical isolates on native plants and *A. adenophora*. AA, *Ageratina adenophora*; AB, *Achyranthes bidentata*; AD, *Ampelopsis delavayana*; AE, *Amphicarpaea edgeworthii*; AH, *Arthraxon hispidus*; AM, *Abelmoschus moschatus*; AN, *Alnus nepalensis*; BE, *Bambusa emeiensis*; CG, *Cyclobalanopsis glauca*; CO, *Cynanchum otophyllum*; CT, *Celtis tetrandra*; FM, *Fallopia multiflora*; HT, *Hypoestes triflora*; LC, *Lindera communis*; MF, *Michelia figo*; MR, *Myrica rubra*; OF, *Oreocnide frutescens*; PH, *Petunia hybrida*; PP, *Pueraria peduncularis*; PT, *Parthenocissus tricuspidata*; QV, *Quercus variabilis*; RC, *Rubia cordifolia*; RI, *Reinwardtia indica*; RP, *Rubus parvifolius*; RX, *Rosa xanthina*; SE, *Sechium edule*; SS, *Smilax scobinicaulis*; UL, *Urena lobata*; ZB, *Zanthoxylum bungeanum*; and ZM, *Zehneria maysorensis.* Isolate names in red represent fungi that are highly virulent toward *A. adenophora*. Plant names in red represent the tested hosts from Figure 2.

For 18 isolates with high virulence against *A. adenophora,* we expanded the virulence test to a further 15 native plants (Figure 5A). Again, compared with other genera, *Epicoccum* exerted the strongest virulence and had the widest host range. Three isolates—S188 (*M. speciosa*), G56 (*M. ageratinae*), and Y122 (*M. ageratinae*)—displayed strong virulence toward *A. adenophora* but were only weakly virulent toward native plants and had a narrow host range (Figure 5B).

### Evaluation of Disease Development on the Leaves of *Ageratina adenophora* With Different Invasion Histories

**Figure 6:**
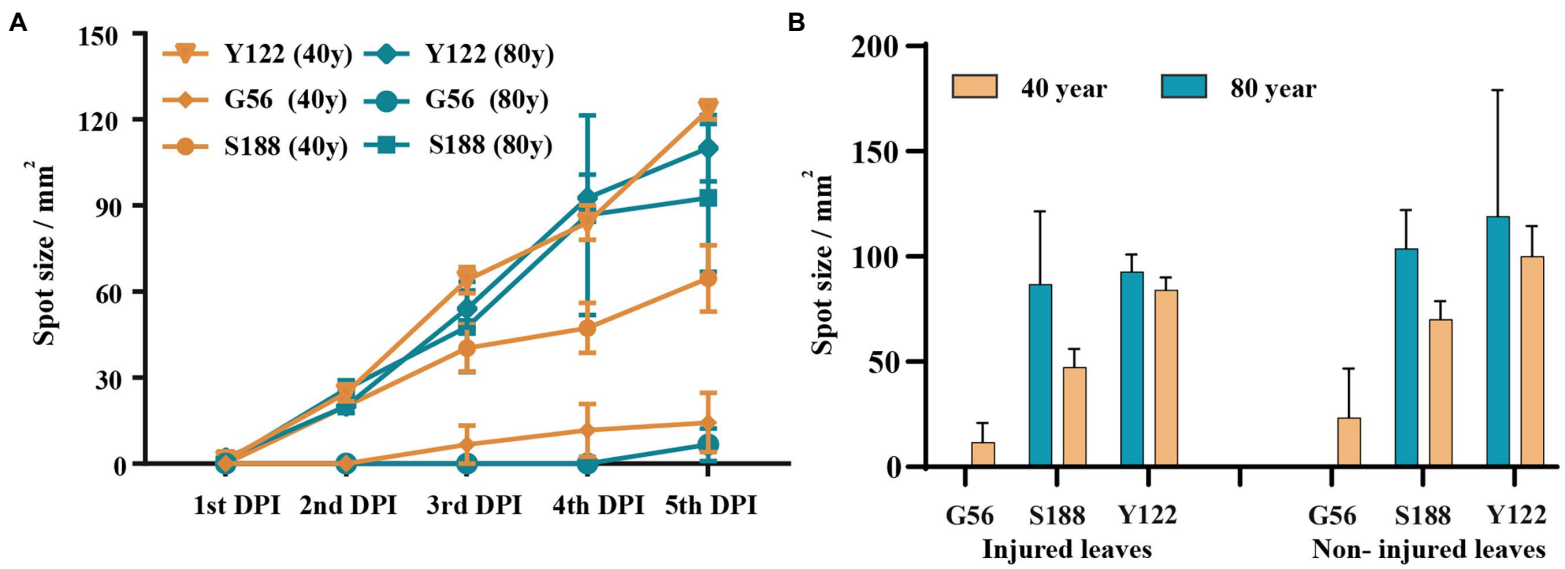
Virulence evaluation for potential biocontrol strains. **(A)** The course of disease development on injured leaves. **(B)** Comparison of lesion sizes at day 4 post-inoculation between injured and non-injured leaves from *Ageratina adenophora* with different invasion histories. There were significant differences in leaf spot size among the isolates of the injured leaves of *A. adenophora* with an 80-year invasion history (*p* = 0.030, Kruskal-Wallis test). Error bars represent 1 SE.

Using detached leaves, three isolates—S188 (*M. speciosa*), G56 (*M. ageratinae*), and Y122 (*M. ageratinae*)—were further tested for their virulence toward *A. adenophora* with different invasive histories (Figure 6). Disease development after inoculation with either Y122 or S188 was slightly faster than that for inoculation with G56; however, leaf spot size did not differ for all three isolates (Mann–Whitney U test, *p* > 0.05) (Figure 6A). On average, the lesion size of uninjured leaves was even slightly greater than that of injured leaves; isolates Y122 and S188 exerted stronger virulence than G56 (Figure 6B). The spray experiments using seedlings showed that Y122 and S188, but not G56, could cause lesions in the leaves of *A. adenophora* (Supplementary Figure 3).

## Discussion

Increasing evidence has indicated that invasive plants can be infected by diverse pathogens in their introduced range (Mitchell et al., 2010; Flory and Clay, 2013; Stricker et al., 2016). Investigating the pathogen communities associated with invasive hosts is important for understanding pathogen-mediated interactions with invasive plants and co-occurring native plants, as well as for evaluating the long-term effects of disease emergence and pathogen accumulation (Stricker et al., 2016; Chen et al., 2020).

Flory et al. (2011) reported that members of a single fungal genus (*Bipolaris*) represent multiple undescribed species, and many fungi associated with *Ageratina adenophora* are potentially also novel species. Although the taxonomy of the family Didymellaceae was recently comprehensively evaluated (Chen et al., 2017; Valenzuela-Lopez et al., 2018), we nevertheless identified new species within the genus *Remotididymella* (*R. ageratinae* and *R. anemophila*) (Yang et al., 2021). Moreover, strains from *Mesophoma speciosa* and *M. ageratinae* may also represent two novel species from one novel genus (Figure 1A; Supplementary Figures 1, 2; Yang et al., unpublished data). The reasons why invasive plants are likely to accumulate undescribed species may be ascribed to (1) that fungi/invasive plant interactions are rarely investigated; (2) the amplification of previously rare fungi by invasive plants in the introduced range; and (3) fungi co-introduced by invasive plants from the native range are rarely investigated. This suggests that invasive plants may be an important source as yet unidentified microbial species, including potential pathogens.

The “accumulation of local pathogens” hypothesis stipulates that pathogens accumulated on invasive alien plants may spread to native plants and indirectly enhance the competitive advantage of the former in the cases where the alien species are more tolerant to pathogens than the native plants (Eppinga et al., 2006). For example, the invasive weed *Chromolaena odorata* can accumulate high concentrations of the generalist soil-borne fungal pathogen *Fusarium semitectum* in their invaded range, thereby negatively affecting native plant species (Mangla and Callaway, 2007; Kowalski et al., 2015). In our study, the probability of *A. adenophora* gaining a competitive advantage over native plants through these Didymellaceae fungi was low as, in general, these fungi are more virulent toward *A. adenophora* than to native plants (Figure 2). In particular, isolates of *Boeremia exigua*, *Epicoccum latusicollum*, *E. sorghinum*, *M. speciosa*, and *M. ageratinae* exerted strong virulence against *A. adenophora* (Figure 2). Accordingly, in the long-term, the accumulated pathogens are likely to slow down the expansion of invasion and even stop the expansion of *A. adenophora*, in line with that previously described (Hilker et al., 2005; Flory et al., 2011).

Our findings suggested that *A. adenophora* may serve as a reservoir of pathogens for native plants in invaded ecosystems. We have previously reported that there was extensive overlap between the foliar fungi of *A. adenophora* and the pathogens of native plants (Chen et al., 2020). In this study, *Epicoccum,* a widespread pathogen (Chen et al., 2017; Zhou et al., 2018; Lin and Hand, 2019), was isolated from both *A. adenophora* and native plants (Figure 2) and most of the isolates were found to be strongly virulent against *A. adenophora* as well as a variety of native plants (Figure 3). Similarly, isolates of *Didymella*, a common plant pathogen of crops (Huang et al., 2018; Moral et al., 2018; Ren et al., 2019), were more virulent toward native plants than to *A. adenophora* (Figure 2). Because *A. adenophora* is now widely distributed worldwide (Wang and Wang, 2006), these generalists pose a high risk of disease transmission in invaded ecosystems.

Studies have shown that host-specific fungal pathogens exert negative effects on phylogenetically related neighboring trees (Johnson et al., 2012; Liu et al., 2012; Chen et al., 2019). In this study, ParaFit test analysis indicated that there was no host plant–pathogen co-evolution (Figure 4, *p* > 0.05). The plant–pathogen association network was found to be non-random for all Didymellaceae isolates used in this experiment (Figure 4), indicating that most Didymellaceae isolates had a broad host range in the foliar system. This “shotgun” infection mode further suggests that there is frequent fungal transmission between *A. adenophora* and co-occurring native plants, in line with our previous report (Chen et al., 2020). Interestingly, we did not obtain isolates belonging to *M. speciosa* from native plants (Figure 2, named OTU515 in our previous report, see Chen et al., 2020). That the ITS locus of this Group is highly concurrent with those found in fungal DNA extracted from *A. adenophora* seeds (Fang et al., 2021) suggested that this fungus may co-spread with *A. adenophora* in a seed-borne manner. Nonetheless, the identification of the original source of these fungi requires further investigation, such as a comparison of their abundance and species composition in the seeds of *A. adenophora* in its native and invaded ranges.

*Passalora ageratinae* (Mycosphaerellaceae) and *Baeodromus eupatorii* (Pucciniosiraceae) have been proposed as biological control agents for *A. adenophora* in countries such as Australia, China, South Africa, and the USA, among others (Poudel et al., 2019). In this study, we evaluated the biocontrol potential of pathogens of *A. adenophora* by testing their host range and virulence. Strains from this family (Didymellaceae), and particularly those belonging to the species *E. latusicollum* and *E. sorghinum*, were found to be highly virulent and to also have a wide host range (Figures 3, 5). In contrast, *M. speciosa* and *M. ageratinae* exerted greater virulence against *A. adenophora* than toward native plants, and this was particularly true for Y122 (*M. ageratinae*) and S188 (*M. speciosa*), which also had relatively narrow host ranges. Although these observations highlight the potential of these strains as biocontrol agents for *A. adenophora*, the pathogenicity test showed that these isolates could also infect some native plants (Figures 2, 4). Further investigation is needed to improve these strains for biocontrol, including a detailed genomic and transcriptomic analysis (Massonnet et al., 2018; Guo et al., 2020; Sarsaiya et al., 2021).

## Data Availability Statement

The datasets presented in this study can be found in online repositories. The names of the repository/repositories and accession number(s) can be found at: National Center for Biotechnology Information (NCBI) under accession numbers OM698599-OM698811 (LSU), OM698383-OM698595 (ITS1), OM751626-OM751838 (tub2), and OM751415-OM751625 (rpb2).

## Author Contributions

H-BZ designed the study and revised the manuscript. LC, A-LY, and Y-XL performed the sample collection, molecular identification, and virulence tests. LC performed the statistical analysis and prepared the manuscript. All authors contributed to the article and approved the submitted version.

## Funding

This study was supported financially by grants from the Major Science and Technology Project in Yunnan Province, China (no. K264202011020) and the National Natural Science Foundation of China (grant no. 31770585) to H-BZ. The funders had no role in the study design, data collection and analysis, decision to publish, or preparation of the manuscript.

## Conflict of Interest

The authors declare that the research was conducted in the absence of any commercial or financial relationships that could be construed as a potential conflict of interest.

## Publisher’s Note

All claims expressed in this article are solely those of the authors and do not necessarily represent those of their affiliated organizations, or those of the publisher, the editors and the reviewers. Any product that may be evaluated in this article, or claim that may be made by its manufacturer, is not guaranteed or endorsed by the publisher.

## References

[ref1] Arnold A. E. Lutzoni F. (2007). Diversity and host range of foliar fungal endophytes: are tropical leaves biodiversity hotspots? Ecology 88, 541–549. doi: 10.1890/05-1459, PMID: 17503580

[ref2] Beckstead J. Meyer S. E. Connolly B. M. Huck M. B. Street L. E. (2010). Cheatgrass facilitates spillover of a seed bank pathogen onto native grass species. J. Ecol. 98, 168–177. doi: 10.1111/j.1365-2745.2009.01599.x

[ref3] Buccellato L. Fisher J. T. Witkowski E. T. F. Byrne M. J. (2021). The effects of a stem gall fly and a leaf pathogen on the reproductive output of Crofton weed, *Ageratina adenophora* (Asteraceae), in greenhouse and field trials. Biol. Control 152:104453. doi: 10.1016/j.biocontrol.2020.104453

[ref4] Bufford J. L. Hulme P. E. Sikes B. A. Cooper J. A. Johnston P. R. Duncan R. P. (2016). Taxonomic similarity, more than contact opportunity, explains novel plant-pathogen associations between native and alien taxa. New Phytol. 212, 657–667. doi: 10.1111/nph.14077, PMID: 27440585

[ref5] Chen Q. Hou L. W. Duan W. J. Crous P. W. Cai L. (2017). Didymellaceae revisited. Stud. Mycol. 87, 105–159. doi: 10.1016/j.simyco.2017.06.002, PMID: 28706324 PMC5498420

[ref6] Chen Y. Jia P. Cadotte M. W. Wang P. Liu X. Qi Y.. (2019). Rare and phylogenetically distinct plant species exhibit less diverse root-associated pathogen communities. J. Ecol. 107, 1226–1237. doi: 10.1111/1365-2745.13099

[ref7] Chen Q. Zhang K. Zhang G. Z. Cai L. (2015). A polyphasic approach to characterise two novel species of Phoma (Didymellaceae) from China. Phytotaxa 197, 267–281. doi: 10.11646/phytotaxa.197.4.4

[ref8] Chen L. Zhou J. Zeng T. Miao Y.-F. Mei L. Yao G.-B.. (2020). Quantifying the sharing of foliar fungal pathogens by the invasive plant *Ageratina adenophora* and its neighbours. New Phytol. 227, 1493–1504. doi: 10.1111/nph.16624, PMID: 32343409

[ref9] Colautti R. I. Ricciardi A. Grigorovich I. A. MacIsaac H. J. (2004). Is invasion success explained by the enemy release hypothesis? Ecol. Lett. 7, 721–733. doi: 10.1111/j.1461-0248.2004.00616.x

[ref10] Dickie I. A. Bufford J. L. Cobb R. C. Desprez-Loustau M.-L. Grelet G. Hulme P. E.. (2017). The emerging science of linked plant–fungal invasions. New Phytol. 215, 1314–1332. doi: 10.1111/nph.14657, PMID: 28649741

[ref11] Eppinga M. Rietkerk M. Dekker S. Ruiter P. Putten W. (2006). Accumulation of local pathogens: a new hypothesis to explain exotic plant invasions. Oikos 114, 168–176. doi: 10.1111/j.2006.0030-1299.14625.x

[ref12] Fang K. Chen L. Zhou J. Yang Z. P. Dong X. F. Zhang H. B. (2019). Plant–soil–foliage feedbacks on seed germination and seedling growth of the invasive plant *Ageratina adenophora*. Proc. Roy. Soc. B-Biol. Sci. 286:20191520. doi: 10.1098/rspb.2019.1520, PMID: 31822255 PMC6939910

[ref13] Fang K. Zhou J. Chen L. Li Y. X. Yang A. L. Dong X. F.. (2021). Virulence and community dynamics of fungal species with vertical and horizontal transmission on a plant with multiple infections. PLoS Pathog. 17:e1009769. doi: 10.1371/journal.ppat.1009769, PMID: 34265026 PMC8315517

[ref14] Flory S. L. Alba C. Clay K. Holt R. D. Goss E. M. (2018). Emerging pathogens can suppress invaders and promote native species recovery. Biol. Invasions 20, 5–8. doi: 10.1007/s10530-017-1438-9

[ref15] Flory S. L. Clay K. (2013). Pathogen accumulation and long-term dynamics of plant invasions. J. Ecol. 101, 607–613. doi: 10.1111/1365-2745.12078

[ref16] Flory S. L. Kleczewski N. Clay K. (2011). Ecological consequences of pathogen accumulation on an invasive grass. Ecosphere 2:art120. doi: 10.1890/ES11-00191.1

[ref17] Gilbert G. S. Webb C. O. (2007). Phylogenetic signal in plant pathogen-host range. Proc. Natl. Acad. Sci. U. S. A. 104, 4979–4983. doi: 10.1073/pnas.0607968104, PMID: 17360396 PMC1829250

[ref18] Guo R. Wang Z. Zhou C. Liu Z. Zhang P. Fan H. (2020). Transcriptomic analysis reveals biocontrol mechanisms of Trichoderma harzianum ACCC30371 under eight culture conditions. J. Forestry Res. 31, 1863–1873. doi: 10.1007/s11676-019-00912-1

[ref19] Hall T. (1999). BioEdit: a user-friendly biological sequence alignment editor and analysis program for windows 95/98/NT. Nucl. Acids. Symp. Se*r.* 41, 95–98.

[ref20] Hilker F. M. Lewis M. A. Seno H. Langlais M. Malchow H. (2005). Pathogens can slow down or reverse invasion fronts of their hosts. Biol. Invasions 7, 817–832. doi: 10.1007/s10530-005-5215-9

[ref21] Hou L. W. Groenewald J. Z. Pfenning L. H. Yarden O. Crous P. W. Cai L. (2020). The phoma-like dilemma. Stud. Mycol. 96, 309–396. doi: 10.1016/j.simyco.2020.05.001, PMID: 32904212 PMC7452269

[ref22] Huang S. L. Wang L. Wang T. Jiao Z. J. Pang F. H. Tao A. L.. (2018). First report of Didymella leaf blight on Cornus officinalis caused by Didymella glomerata in China. Plant Dis. 102:1031. doi: 10.1094/PDIS-07-17-0933-PDN

[ref23] Institute of Botany (2019). Flora of China.The Chinese Academy of Sciences. Available at: http://www.iplant.cn/foc (Accessed April 5, 2022).

[ref24] Jiang H. Shi Y. T. Zhou Z. X. Yang C. Chen Y. J. Chen L. M.. (2011). Leaf chemistry and co-occurring species interactions affecting the endophytic fungal composition of Eupatorium adenophorum. Ann. Microbiol. 61, 655–662. doi: 10.1007/s13213-010-0186-1

[ref25] Johnson D. J. Beaulieu W. T. Bever J. D. Clay K. (2012). Conspecific negative density dependence and forest diversity. Science 336, 904–907. doi: 10.1126/science.1220269 22605774

[ref26] Keane R. M. Crawley M. J. (2002). Exotic plant invasions and the enemy release hypothesis. Trends Ecol. Evol. 17, 164–170. doi: 10.1016/S0169-5347(02)02499-0

[ref27] Kleczewski N. M. Flory S. L. Clay K. (2012). Variation in pathogenicity and host range of Bipolaris sp causing leaf blight disease on the invasive grass Microstegium vimineum. Weed Sci. 60, 486–493. doi: 10.1614/WS-D-11-00187.1

[ref28] Kolde R. (2018). Pheatmap: Pretty Heatmaps. R Package V. 1.0.10. [WWW document] [Online]. Available at: https://CRAN.R-project.org/package=pheatmap (Accessed April 12, 2022).

[ref29] Kowalski K. Bacon C. Bickford W. Braun H. Clay K. Leduc-Lapierre M.. (2015). Advancing the science of microbial symbiosis to support invasive species management: a case study on Phragmites in the Great Lakes. Front. Microbiol. 6:95. doi: 10.3389/fmicb.2015.00095, PMID: 25745417 PMC4333861

[ref30] Legendre P. Desdevises Y. Bazin E. (2002). A statistical test for host-parasite coevolution. Syst. Biol. 51, 217–234. doi: 10.1080/10635150252899734, PMID: 12028729

[ref31] Lin S. Hand F. P. (2019). Investigations on the timing of fruit infection by fungal pathogens causing fruit rot of deciduous Holly. Plant Dis. 103, 308–314. doi: 10.1094/PDIS-06-18-0973-RE, PMID: 30522396

[ref32] Liu X. Liang M. Etienne R. S. Wang Y. Staehelin C. Yu S. (2012). Experimental evidence for a phylogenetic Janzen-Connell effect in a subtropical forest. Ecol. Lett. 15, 111–118. doi: 10.1111/j.1461-0248.2011.01715.x, PMID: 22082078

[ref33] Liu Y. J. J. Whelen S. Benjamin D. H. (1999). Phylogenetic relationships among ascomycetes: evidence from an RNA polymerase II subunit. Mol. Biol. Evol. 16, 1799–1808. doi: 10.1093/oxfordjournals.molbev.a026092, PMID: 10605121

[ref34] Mangla S. Callaway R. (2007). Exotic invasive plant accumulates native soil pathogens which inhibit native plants. J. Ecol. 96, 58–67. doi: 10.1111/j.1365-2745.2007.01312.x

[ref35] Massonnet M. Morales-Cruz A. Minio A. Figueroa-Balderas R. Lawrence D. P. Travadon R.. (2018). Whole-genome resequencing and pan-transcriptome reconstruction highlight the impact of genomic structural variation on secondary metabolite gene clusters in the grapevine Esca pathogen Phaeoacremonium minimum. Front. Microbiol. 9:1784. doi: 10.3389/fmicb.2018.01784, PMID: 30150972 PMC6099105

[ref36] Mitchell C. E. Blumenthal D. Jarošík V. Puckett E. E. Pyšek P. (2010). Controls on pathogen species richness in plants' introduced and native ranges: roles of residence time, range size and host traits. Ecol. Lett. 13, 1525–1535. doi: 10.1111/j.1461-0248.2010.01543.x, PMID: 20973907 PMC3003901

[ref37] Moral J. Lichtemberg P. S. F. Papagelis A. Sherman J. Michailides T. J. (2018). Didymella glomerata causing leaf blight on pistachio. Eur. J. Plant Pathol. 151, 1095–1099. doi: 10.1007/s10658-018-1422-y

[ref38] Murray M. G. Thompson W. F. (1980). Rapid isolation of high molecular weight plant DNA. Nucleic Acids Res. 8, 4321–4326. doi: 10.1093/nar/8.19.4321, PMID: 7433111 PMC324241

[ref39] Poudel A. S. Jha P. K. Shrestha B. B. Muniappan R. (2019). Biology and management of the invasive weed *Ageratina adenophora* (Asteraceae): current state of knowledge and future research needs. Weed Res. 59, 79–92. doi: 10.1111/wre.12351

[ref40] Purse B. V. Graeser P. Searle K. Edwards C. Harris C. (2013). Challenges in predicting invasive reservoir hosts of emerging pathogens: mapping *Rhododendron ponticum* as a foliar host for *Phytophthora ramorum* and *Phytophthora kernoviae* in the UK. Biol. Invasions 15, 529–545. doi: 10.1007/s10530-012-0305-y

[ref41] Ren Y. F. Li D. X. Zhao X. Z. Wang Y. Bao X. T. Wang X.. (2019). Whole genome sequences of the tea leaf spot pathogen Didymella segeticola. Phytopathology 109, 1676–1678. doi: 10.1094/PHYTO-02-19-0050-A, PMID: 31188072

[ref42] Ronquist F. Teslenko M. Mark P. V. D. Ayres D. L. Darling A. Höhna S.. (2012). MrBayes 3.2: efficient Bayesian phylogenetic inference and model choice across a large model space. Syst. Biol. 61, 539–542. doi: 10.1093/sysbio/sys029, PMID: 22357727 PMC3329765

[ref43] Sarsaiya S. Jain A. Shi J. Chen J. (2021). “1 - fungi endophytes for biofactory of secondary metabolites: genomics and metabolism,” in Biocontrol Agents and Secondary Metabolites. ed. Jogaiah S. (Sawston: Woodhead Publishing), 1–21.

[ref44] Stamatakis A. (2014). RAxML version 8: a tool for phylogenetic analysis and post-analysis of large phylogenies. Bioinformatics 30, 1312–1313. doi: 10.1093/bioinformatics/btu033, PMID: 24451623 PMC3998144

[ref45] Stricker K. B. Harmon P. F. Goss E. M. Clay K. Flory S. L. (2016). Emergence and accumulation of novel pathogens suppress an invasive species. Ecol. Lett. 19, 469–477. doi: 10.1111/ele.12583, PMID: 26931647

[ref46] Sung G.-H. Sung J.-M. Hywel-Jones N. L. Spatafora J. W. (2007). A multi-gene phylogeny of Clavicipitaceae (Ascomycota, fungi): identification of localized incongruence using a combinational bootstrap approach. Mol. Phylogenet. Evol. 44, 1204–1223. doi: 10.1016/j.ympev.2007.03.011, PMID: 17555990

[ref47] Valenzuela-Lopez N. Cano-Lira J. F. Guarro J. Sutton D. A. Wiederhold N. Crous P. W.. (2018). Coelomycetous Dothideomycetes with emphasis on the families Cucurbitariaceae and Didymellaceae. Stud. Mycol. 90, 1–69. doi: 10.1016/j.simyco.2017.11.003, PMID: 29255336 PMC5725287

[ref48] Vilgalys R. Hester M. (1990). Rapid genetic identification and mapping of enzymatically amplified ribosomal DNA from several Cryptococcus species. J. Bacteriol. 172, 4238–4246. doi: 10.1128/jb.172.8.4238-4246.1990, PMID: 2376561 PMC213247

[ref49] Wan Z. X. Zhu J. J. Qiang S. (2001). The pathogenic mechanism of toxin of *Alternaria alternata* (Fr.) Keissler to *Eupatorium adenophorum*. J. Plant Resour. Environ. 10, 47–50 (In Chinese).

[ref50] Wang C. Lin H. L. Feng Q. S. Jin C. Y. Cao A. C. He L. (2017). A new strategy for the prevention and control of Eupatorium adenophorum under climate change in China. Sustainability 9:11. doi: 10.3390/su9112037

[ref51] Wang R. Wang Y. Z. (2006). Invasion dynamics and potential spread of the invasive alien plant species *Ageratina adenophora* (Asteraceae) in China. Divers. Distrib. 12, 397–408. doi: 10.1111/j.1366-9516.2006.00250.x

[ref52] White T. J. Bruns T. Lee S. Taylor J. (1990). In PCR Protocols: A Guide to Methods and Applications. San Diego: Academic Press.

[ref53] Woudenberg J. H. C. Aveskamp M. M. de Gruyter J. Spiers A. G. Crous P. W. (2009). Multiple Didymella teleomorphs are linked to the Phoma clematidina morphotype. Persoonia 22, 56–62. doi: 10.3767/003158509x427808, PMID: 20198138 PMC2789541

[ref54] Yang A.-L. Chen L. Fang K. Dong X.-F. Li Y.-X. Zhang H.-B.. (2021). Remotididymella ageratinae sp. nov. and Remotididymella anemophila sp. nov., two novel species isolated from the invasive weed *Ageratina adenophora* in PR China. Int. J. Syst. Evol. Micr. 71:004572. doi: 10.1099/ijsem.0.004572, PMID: 33206031

[ref01] Yang A.-L. Chen L. Cheng L. Li J.-P. Zeng H.-B. Zhang H.-B.. (2022). Two novel species of Mesophoma gen. nov. from PR China and a potential biocontrol of the invasive weed Ageratina Adenophora. pers. comm.

[ref55] Zhou Z. X. Jiang H. Yang C. Yang M. Z. Zhang H. B. (2010). Microbial community on healthy and diseased leaves of an invasive plant *Eupatorium adenophorum* in Southwest China. J. Microbiol. 48, 139–145. doi: 10.1007/s12275-010-9185-y, PMID: 20437143

[ref56] Zhou H. Liu P. P. Qiu S. Wei S. J. Xia K. Gao Q. (2018). Identity of Epicoccum sorghinum causing leaf spot disease of Bkfilla striata in China. Plant Dis. 102:1039. doi: 10.1094/PDIS-11-17-1757-PDN

