# Supplementary material for: Virulence and Host Range of Fungi Associated With the Invasive Plant Ageratina adenophora
Source: Front Microbiol. 2022 Apr 26;13:857796. doi: 10.3389/fmicb.2022.857796 (PMC9087049; doi:10.3389/fmicb.2022.857796)
Supplement: Supplementary file 1 [file Data_Sheet_1.PDF]

Supplementary Material

1. Supplementary Figures

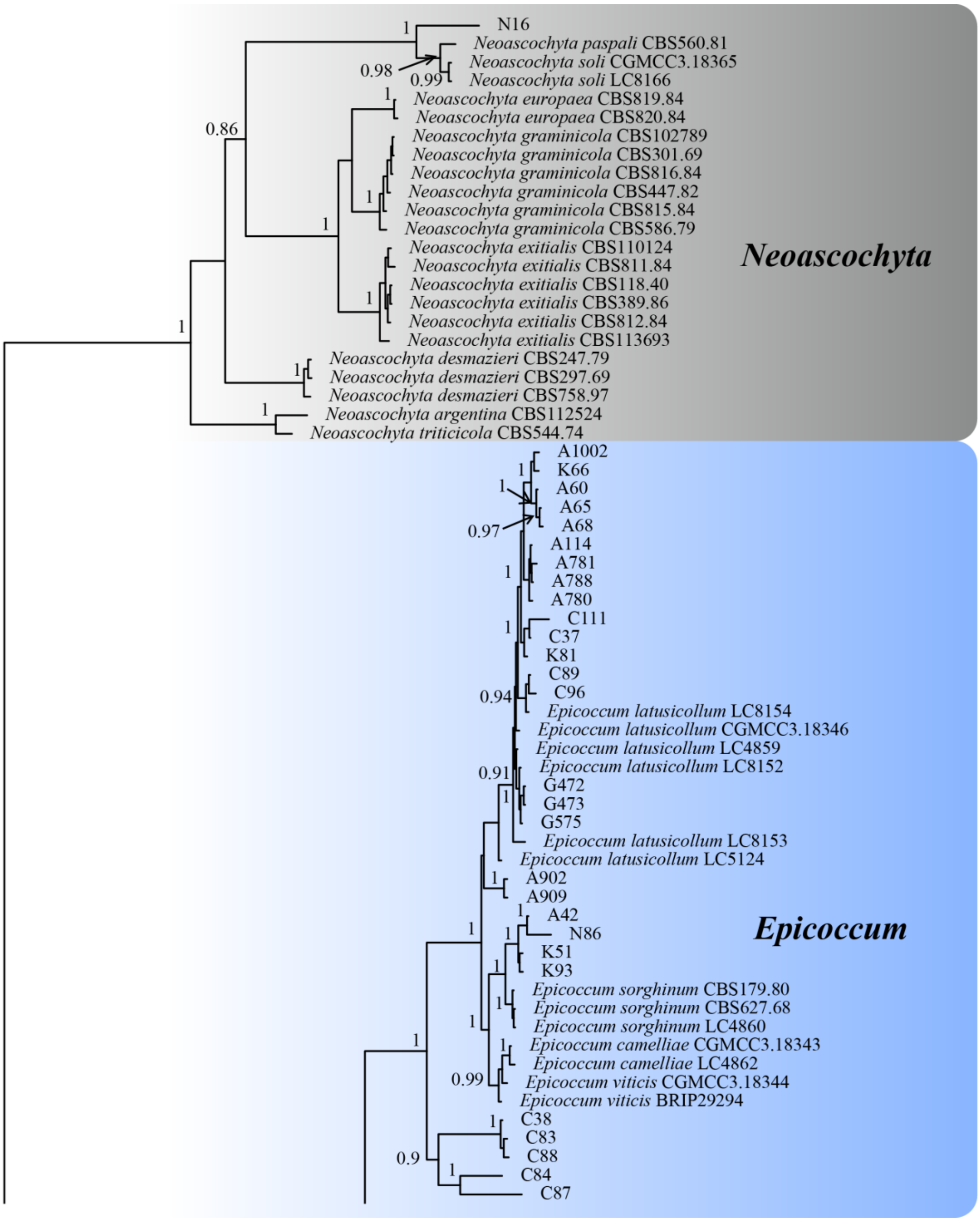

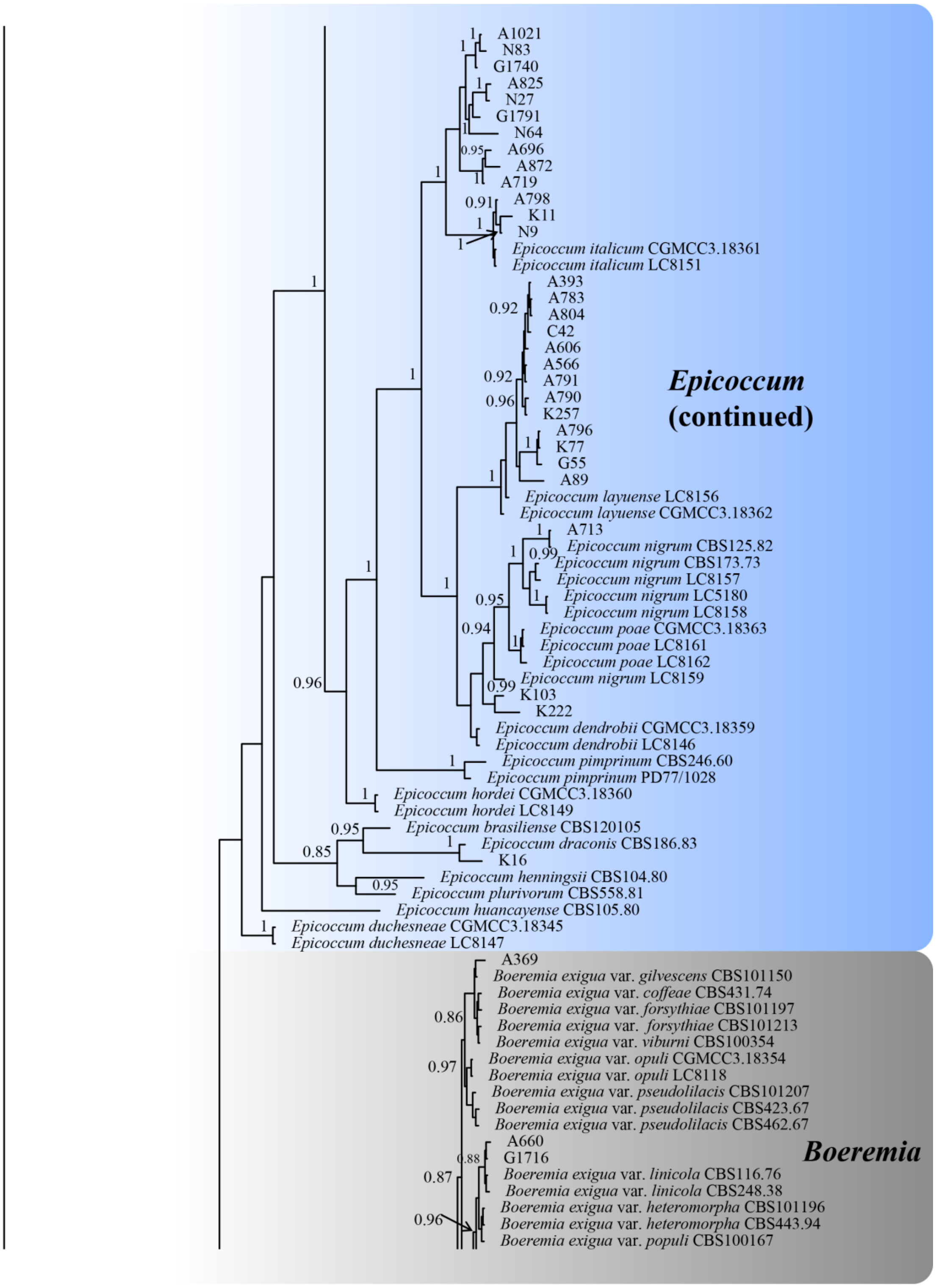
A1021
N83
G1740
A825
N27
G1791
N64
A696
A872
A719
A798
K11
N9
Epicoccum italicum CGMCC3.18361
Epicoccum italicum LC8151
A393
A783
A804
C42
A606
A566
A791
A790
K257
A796
K77
G55
A89
Epicoccum layuense LC8156
Epicoccum layuense CGMCC3.18362
A713
Epicoccum nigrum CBS125.82
Epicoccum nigrum CBS173.73
Epicoccum nigrum LC8157
Epicoccum nigrum LC5180
Epicoccum nigrum LC8158
Epicoccum poae CGMCC3.18363
Epicoccum poae LC8161
Epicoccum poae LC8162
Epicoccum nigrum LC8159
K103
K222
Epicoccum dendrobii CGMCC3.18359
Epicoccum dendrobii LC8146
Epicoccum pimprinum CBS246.60
Epicoccum pimprinum PD77/1028
Epicoccum hordei CGMCC3.18360
Epicoccum hordei LC8149
Epicoccum brasiliense CBS120105
Epicoccum draconis CBS186.83
K16
Epicoccum henningsii CBS104.80
Epicoccum plurivorum CBS558.81
Epicoccum huancayense CBS105.80
Epicoccum duchesneae CGMCC3.18345
Epicoccum duchesneae LC8147
Epicoccum (continued)
A369
Boeremia exigua var. gilvescens CBS101150
Boeremia exigua var. coffeae CBS431.74
Boeremia exigua var. forsythiae CBS101197
Boeremia exigua var. forsythiae CBS101213
Boeremia exigua var. viburni CBS100354
Boeremia exigua var. opuli CGMCC3.18354
Boeremia exigua var. opuli LC8118
Boeremia exigua var. pseudolilacis CBS101207
Boeremia exigua var. pseudolilacis CBS423.67
Boeremia exigua var. pseudolilacis CBS462.67
A660
G1716
Boeremia exigua var. linicola CBS116.76
Boeremia exigua var. linicola CBS248.38
Boeremia exigua var. heteromorpha CBS101196
Boeremia exigua var. heteromorpha CBS443.94
Boeremia exigua var. populi CBS100167
Boeremia

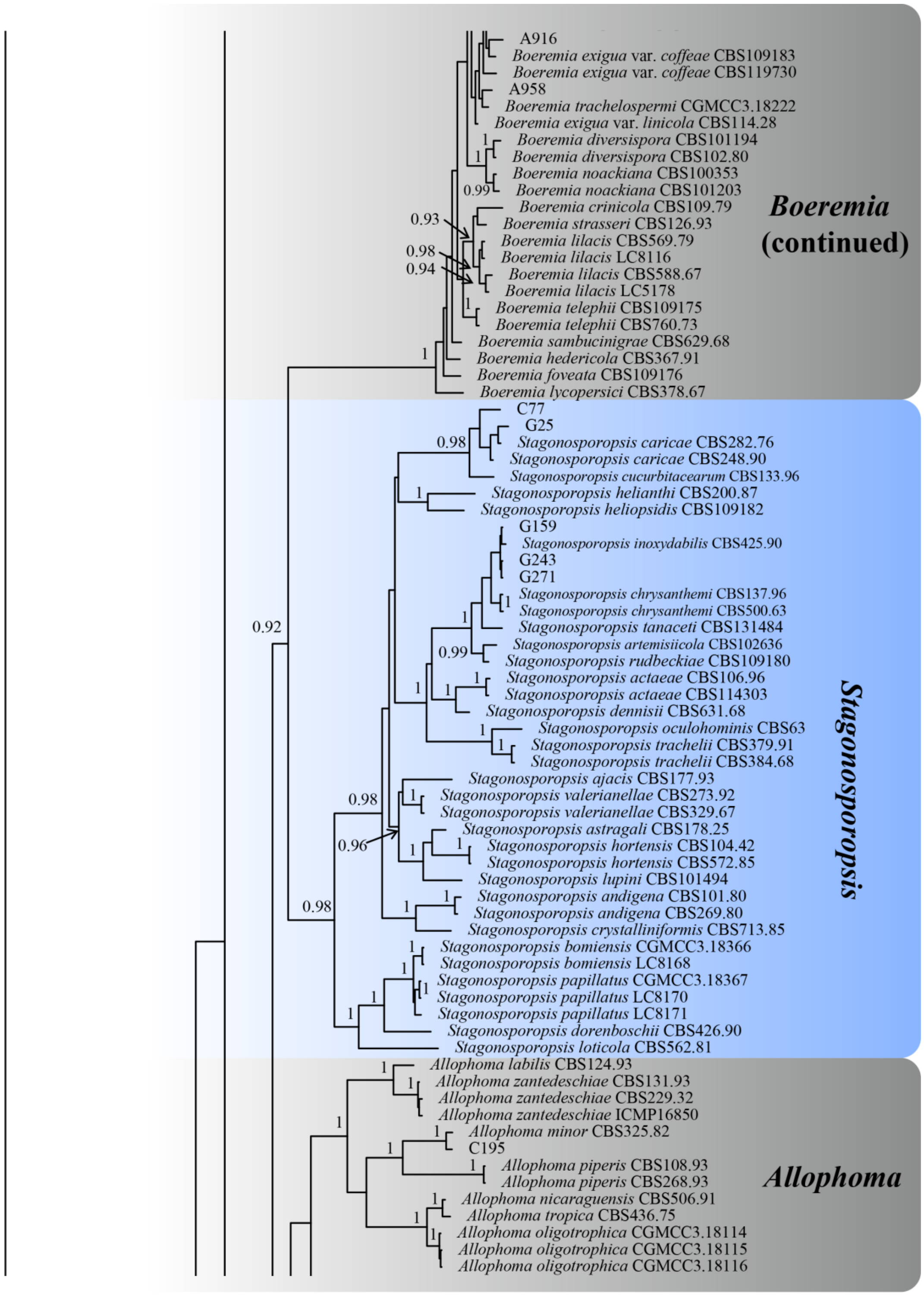
A916
Boeremia exigua var. coffeae CBS109183
Boeremia exigua var. coffeae CBS119730
A958
Boeremia trachelospermi CGMCC3.18222
Boeremia exigua var. linicola CBS114.28
1
Boeremia diversispora CBS101194
1
Boeremia diversispora CBS102.80
Boeremia noackiana CBS100353
0.99
Boeremia noackiana CBS101203
Boeremia crinicola CBS109.79
0.93
Boeremia strasseri CBS126.93
Boeremia lilacis CBS569.79
0.98
Boeremia lilacis LC8116
0.94
Boeremia lilacis CBS588.67
Boeremia lilacis LC5178
1
Boeremia telephii CBS109175
Boeremia telephii CBS760.73
Boeremia sambucinigrae CBS629.68
1
Boeremia hedericola CBS367.91
Boeremia foveata CBS109176
Boeremia lycopersici CBS378.67
Boeremia (continued)
C77
G25
0.98
Stagonosporopsis caricae CBS282.76
Stagonosporopsis caricae CBS248.90
Stagonosporopsis cucurbitacearum CBS133.96
1
Stagonosporopsis helianthi CBS200.87
Stagonosporopsis heliopsidis CBS109182
G159
Stagonosporopsis inoxydabilis CBS425.90
G243
G271
1
Stagonosporopsis chrysanthemi CBS137.96
Stagonosporopsis chrysanthemi CBS500.63
1
Stagonosporopsis tanaceti CBS131484
0.92
0.99
Stagonosporopsis artemisiicola CBS102636
Stagonosporopsis rudbeckiae CBS109180
1
1
Stagonosporopsis actaeae CBS106.96
1
Stagonosporopsis actaeae CBS114303
Stagonosporopsis dennisii CBS631.68
1
Stagonosporopsis oculohominis CBS63
1
Stagonosporopsis trachelii CBS379.91
Stagonosporopsis trachelii CBS384.68
Stagonosporopsis
Stagonosporopsis ajacis CBS177.93
0.98
1
Stagonosporopsis valerianellae CBS273.92
Stagonosporopsis valerianellae CBS329.67
Stagonosporopsis astragali CBS178.25
0.96
1
Stagonosporopsis hortensis CBS104.42
1
Stagonosporopsis hortensis CBS572.85
Stagonosporopsis lupini CBS101494
0.98
1
Stagonosporopsis andigena CBS101.80
1
Stagonosporopsis andigena CBS269.80
Stagonosporopsis crystalliniformis CBS713.85
1
Stagonosporopsis bomiensis CGMCC3.18366
1
Stagonosporopsis bomiensis LC8168
Stagonosporopsis papillatus CGMCC3.18367
1
1
Stagonosporopsis papillatus LC8170
1
Stagonosporopsis papillatus LC8171
Stagonosporopsis dorenboschii CBS426.90
Stagonosporopsis loticola CBS562.81
1
Allophoma labilis CBS124.93
1
Allophoma zantedeschiae CBS131.93
Allophoma zantedeschiae CBS229.32
Allophoma zantedeschiae ICMP16850
1
1
Allophoma minor CBS325.82
1
C195
1
Allophoma piperis CBS108.93
Allophoma piperis CBS268.93
Allophoma
1
Allophoma nicaraguensis CBS506.91
1
Allophoma tropica CBS436.75
1
Allophoma oligotrophica CGMCC3.18114
Allophoma oligotrophica CGMCC3.18115
Allophoma oligotrophica CGMCC3.18116

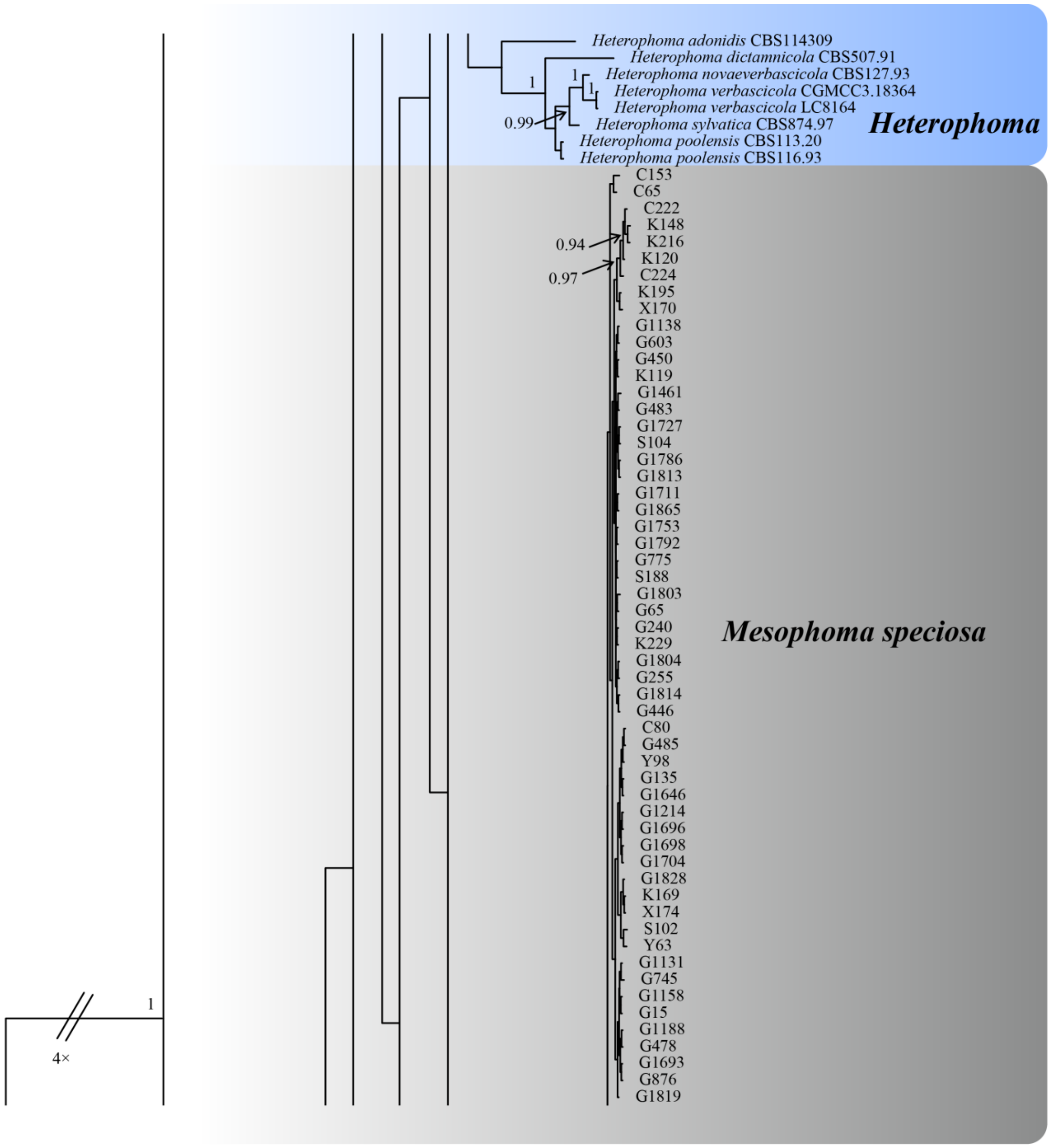
Heterophoma adonidis CBS114309
Heterophoma dictamnicola CBS507.91
Heterophoma novaeverbascicola CBS127.93
Heterophoma verbascicola CGMCC3.18364
Heterophoma verbascicola LC8164
Heterophoma sylvatica CBS874.97
Heterophoma poolensis CBS113.20
Heterophoma poolensis CBS116.93
Heterophoma
0.99
1
1
1
C153
C65
C222
K148
K216
K120
C224
K195
X170
0.94
0.97
G1138
G603
G450
K119
G1461
G483
G1727
S104
G1786
G1813
G1711
G1865
G1753
G1792
G775
S188
G1803
G65
G240
K229
G1804
G255
G1814
G446
C80
G485
Y98
G135
G1646
G1214
G1696
G1698
G1704
G1828
K169
X174
S102
Y63
G1131
G745
G1158
G15
G1188
G478
G1693
G876
G1819
Mesophoma speciosa
4×

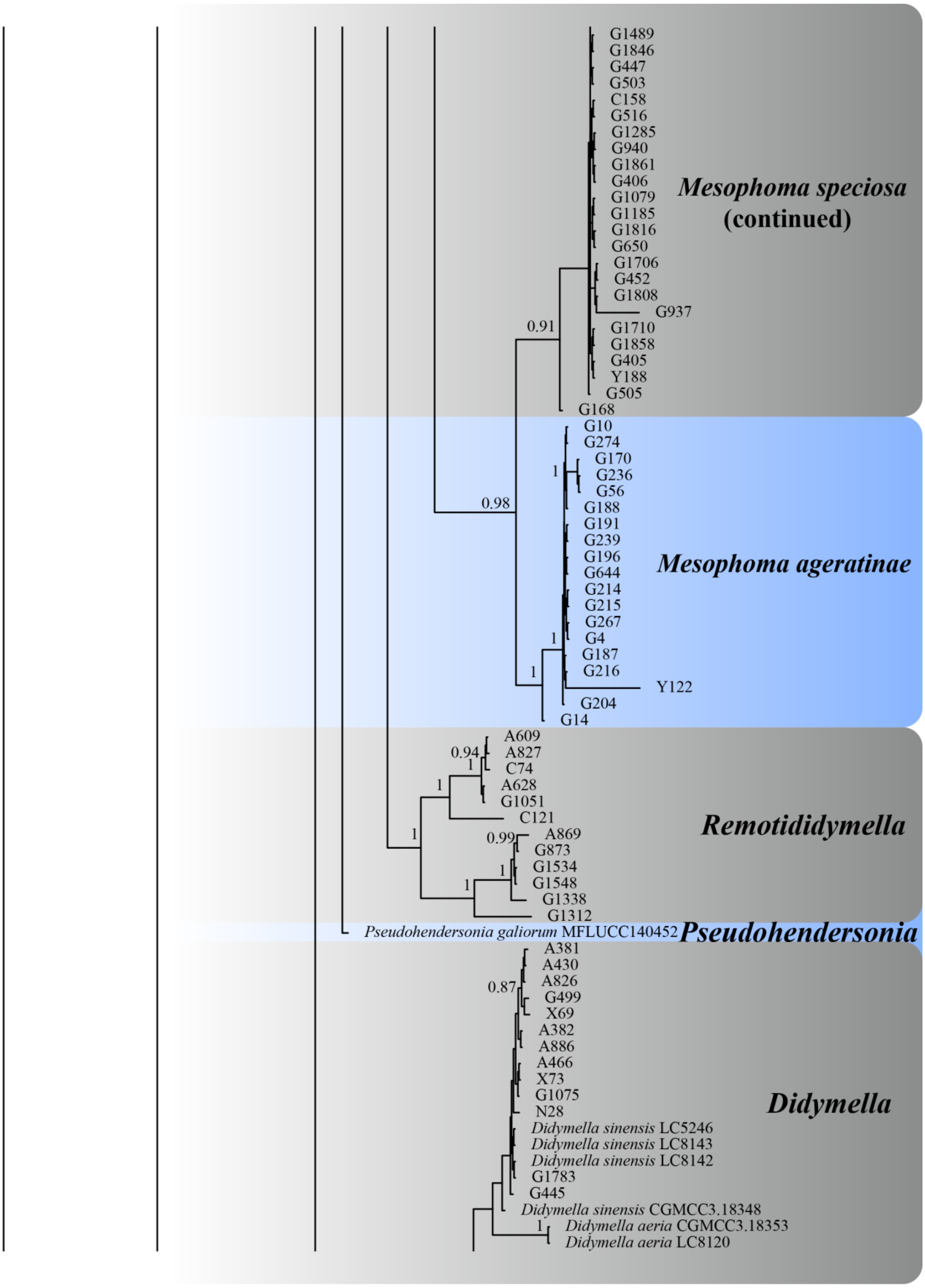
Mesophoma speciosa (continued)
G1489
G1846
G447
G503
C158
G516
G1285
G940
G1861
G406
G1079
G1185
G1816
G650
G1706
G452
G1808
G937
G1710
G1858
G405
Y188
G505
G168
0.91
Mesophoma ageratinae
G10
G274
G170
G236
G56
G188
G191
G239
G196
G644
G214
G215
G267
G4
G187
G216
Y122
G204
G14
0.98
1
1
1
Remotididymella
A609
A827
C74
A628
G1051
C121
A869
G873
G1534
G1548
G1338
G1312
0.94
1
1
1
0.99
1
1
Pseudohendersonia
Pseudohendersonia galiorum MFLUCC140452
Didymella
A381
A430
A826
G499
X69
A382
A886
A466
X73
G1075
N28
Didymella sinensis LC5246
Didymella sinensis LC8143
Didymella sinensis LC8142
G1783
G445
Didymella sinensis CGMCC3.18348
Didymella aeria CGMCC3.18353
Didymella aeria LC8120
0.87
1

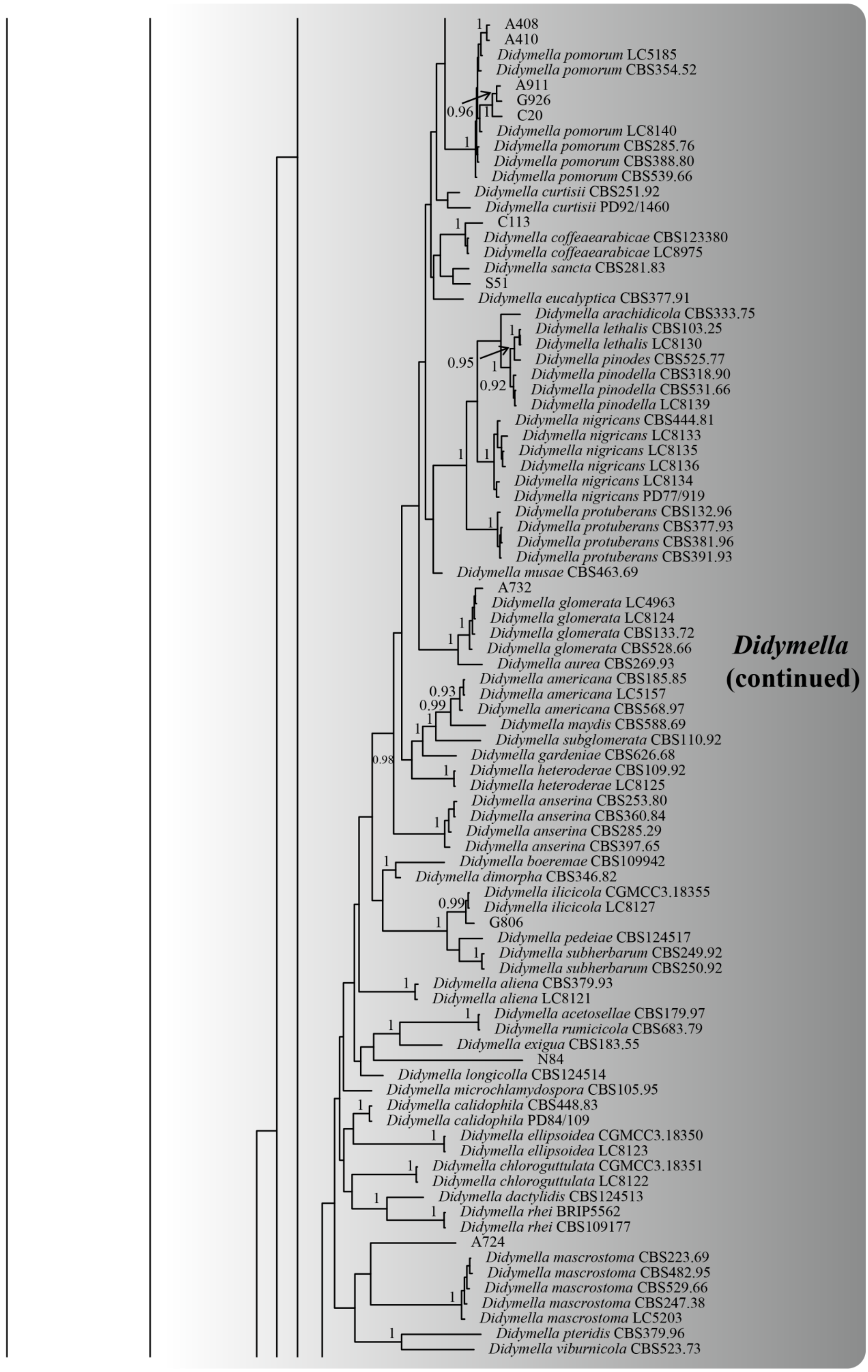
A408
A410
Didymella pomorum LC5185
Didymella pomorum CBS354.52
A911
G926
C20
Didymella pomorum LC8140
Didymella pomorum CBS285.76
Didymella pomorum CBS388.80
Didymella pomorum CBS539.66
Didymella curtisii CBS251.92
Didymella curtisii PD92/1460
C113
Didymella coffeaearabicae CBS123380
Didymella coffeaearabicae LC8975
Didymella sancta CBS281.83
S51
Didymella eucalyptica CBS377.91
Didymella arachidicola CBS333.75
Didymella lethalis CBS103.25
Didymella lethalis LC8130
Didymella pinodes CBS525.77
Didymella pinodella CBS318.90
Didymella pinodella CBS531.66
Didymella pinodella LC8139
Didymella nigricans CBS444.81
Didymella nigricans LC8133
Didymella nigricans LC8135
Didymella nigricans LC8136
Didymella nigricans LC8134
Didymella nigricans PD77/919
Didymella protuberans CBS132.96
Didymella protuberans CBS377.93
Didymella protuberans CBS381.96
Didymella protuberans CBS391.93
Didymella musae CBS463.69
A732
Didymella glomerata LC4963
Didymella glomerata LC8124
Didymella glomerata CBS133.72
Didymella glomerata CBS528.66
Didymella aurea CBS269.93
Didymella americana CBS185.85
Didymella americana LC5157
Didymella americana CBS568.97
Didymella maydis CBS588.69
Didymella subglomerata CBS110.92
Didymella gardeniae CBS626.68
Didymella heteroderae CBS109.92
Didymella heteroderae LC8125
Didymella anserina CBS253.80
Didymella anserina CBS360.84
Didymella anserina CBS285.29
Didymella anserina CBS397.65
Didymella boeremae CBS109942
Didymella dimorpha CBS346.82
Didymella ilicicola CGMCC3.18355
Didymella ilicicola LC8127
G806
Didymella pedeiae CBS124517
Didymella subherbarum CBS249.92
Didymella subherbarum CBS250.92
Didymella aliena CBS379.93
Didymella aliena LC8121
Didymella acetosellae CBS179.97
Didymella rumicicola CBS683.79
Didymella exigua CBS183.55
N84
Didymella longicolla CBS124514
Didymella microchlamydospora CBS105.95
Didymella calidophila CBS448.83
Didymella calidophila PD84/109
Didymella ellipsoidea CGMCC3.18350
Didymella ellipsoidea LC8123
Didymella chloroguttulata CGMCC3.18351
Didymella chloroguttulata LC8122
Didymella dactylidis CBS124513
Didymella rhei BRIP5562
Didymella rhei CBS109177
A724
Didymella mascrostoma CBS223.69
Didymella mascrostoma CBS482.95
Didymella mascrostoma CBS529.66
Didymella mascrostoma CBS247.38
Didymella mascrostoma LC5203
Didymella pteridis CBS379.96
Didymella viburnicola CBS523.73
Didymella
(continued)

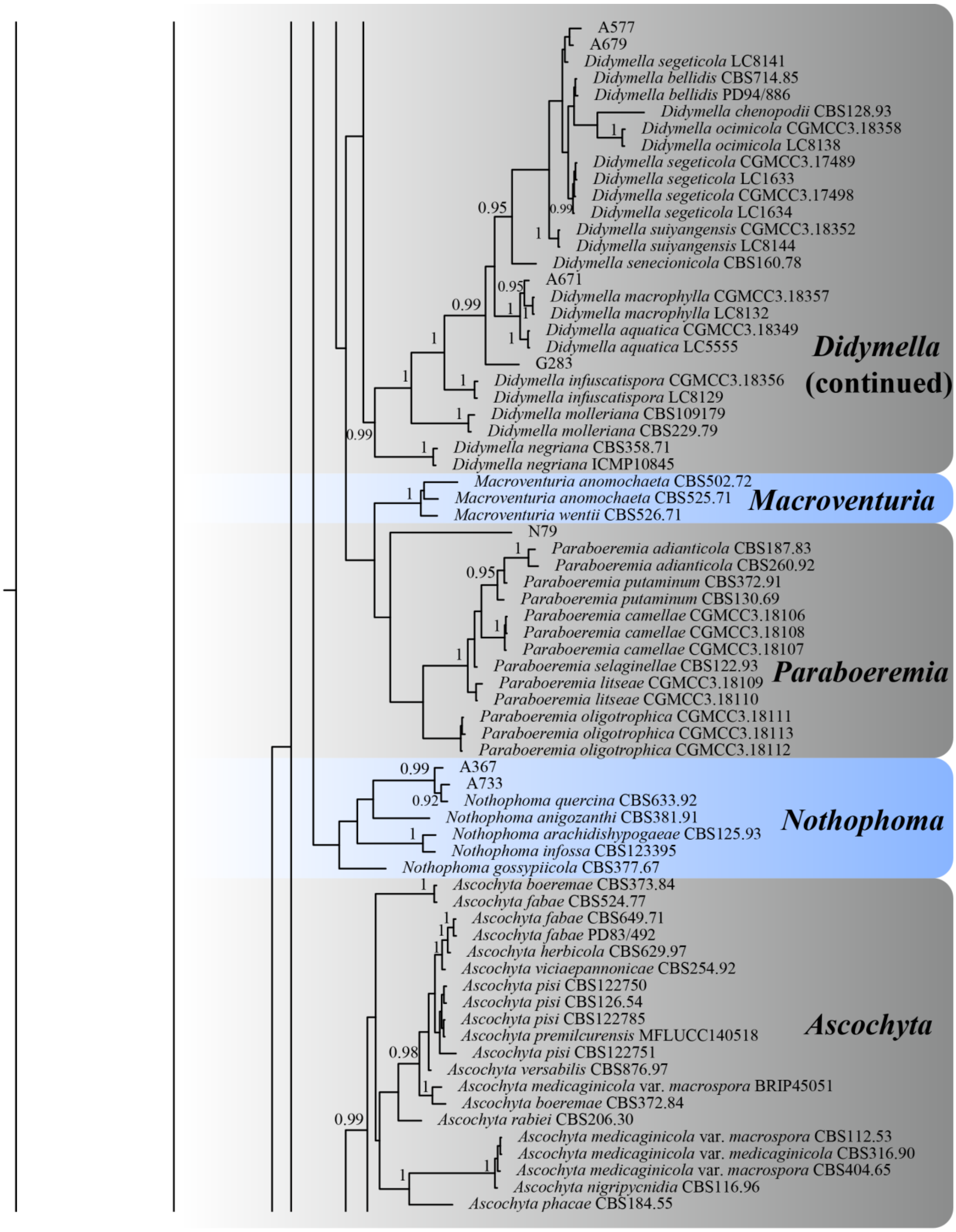

A577
A679
Didymella segeticola LC8141
Didymella bellidis CBS714.85
Didymella bellidis PD94/886
Didymella chenopodii CBS128.93
Didymella ocimicola CGMCC3.18358
Didymella ocimicola LC8138
Didymella segeticola CGMCC3.17489
Didymella segeticola LC1633
Didymella segeticola CGMCC3.17498
Didymella segeticola LC1634
Didymella suiyangensis CGMCC3.18352
Didymella suiyangensis LC8144
Didymella senecionicola CBS160.78
A671
Didymella macrophylla CGMCC3.18357
Didymella macrophylla LC8132
Didymella aquatica CGMCC3.18349
Didymella aquatica LC5555
G283
Didymella infuscatispora CGMCC3.18356
Didymella infuscatispora LC8129
Didymella molleriana CBS109179
Didymella molleriana CBS229.79
Didymella negriana CBS358.71
Didymella negriana ICMP10845
Didymella (continued)
Macroventuria anomochaeta CBS502.72
Macroventuria anomochaeta CBS525.71
Macroventuria wentii CBS526.71
Macroventuria
N79
Paraboeremia adianticola CBS187.83
Paraboeremia adianticola CBS260.92
Paraboeremia putaminum CBS372.91
Paraboeremia putaminum CBS130.69
Paraboeremia camellae CGMCC3.18106
Paraboeremia camellae CGMCC3.18108
Paraboeremia camellae CGMCC3.18107
Paraboeremia selaginellae CBS122.93
Paraboeremia litseae CGMCC3.18109
Paraboeremia litseae CGMCC3.18110
Paraboeremia oligotrophica CGMCC3.18111
Paraboeremia oligotrophica CGMCC3.18113
Paraboeremia oligotrophica CGMCC3.18112
Paraboeremia
A367
A733
Nothophoma quercina CBS633.92
Nothophoma anigozanthi CBS381.91
Nothophoma arachidishypogaeae CBS125.93
Nothophoma infossa CBS123395
Nothophoma gossypiicola CBS377.67
Nothophoma
Ascochyta boeremae CBS373.84
Ascochyta fabae CBS524.77
Ascochyta fabae CBS649.71
Ascochyta fabae PD83/492
Ascochyta herbicola CBS629.97
Ascochyta viciaepannonicae CBS254.92
Ascochyta pisi CBS122750
Ascochyta pisi CBS126.54
Ascochyta pisi CBS122785
Ascochyta premilcurensis MFLUCC140518
Ascochyta pisi CBS122751
Ascochyta versabilis CBS876.97
Ascochyta medicaginicola var. macrospora BRIP45051
Ascochyta boeremae CBS372.84
Ascochyta rabiei CBS206.30
Ascochyta medicaginicola var. macrospora CBS112.53
Ascochyta medicaginicola var. medicaginicola CBS316.90
Ascochyta medicaginicola var. macrospora CBS404.65
Ascochyta nigripycnidia CBS116.96
Ascochyta phacae CBS184.55
Ascochyta
0.95
0.99
0.95
0.99
1
1
0.99
0.95
0.99
0.92
0.98
0.99

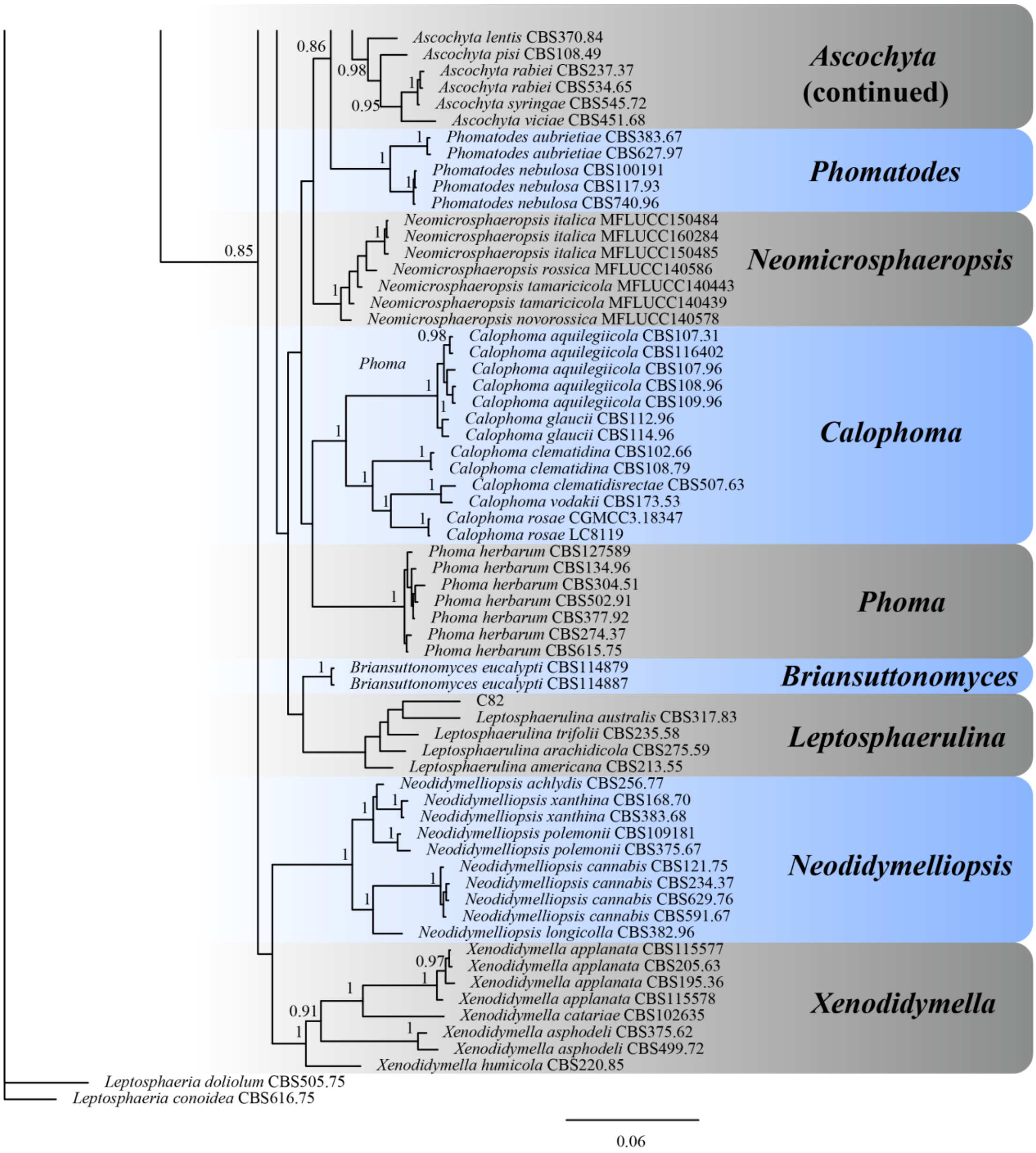

Supplementary Figure 1. Phylogenetic tree inferred from a Bayesian analysis based on a concatenated alignment of LSU, ITS, *rpb2*, and *tub2* sequences of representative strains from all Didymellaceae species based on 21 known genera. The Bayesian posterior probabilities (BPP) are given at the nodes. To adapt the figure to the page, some branches were shortened (shown with a double slash) and marked its shortened multiple.

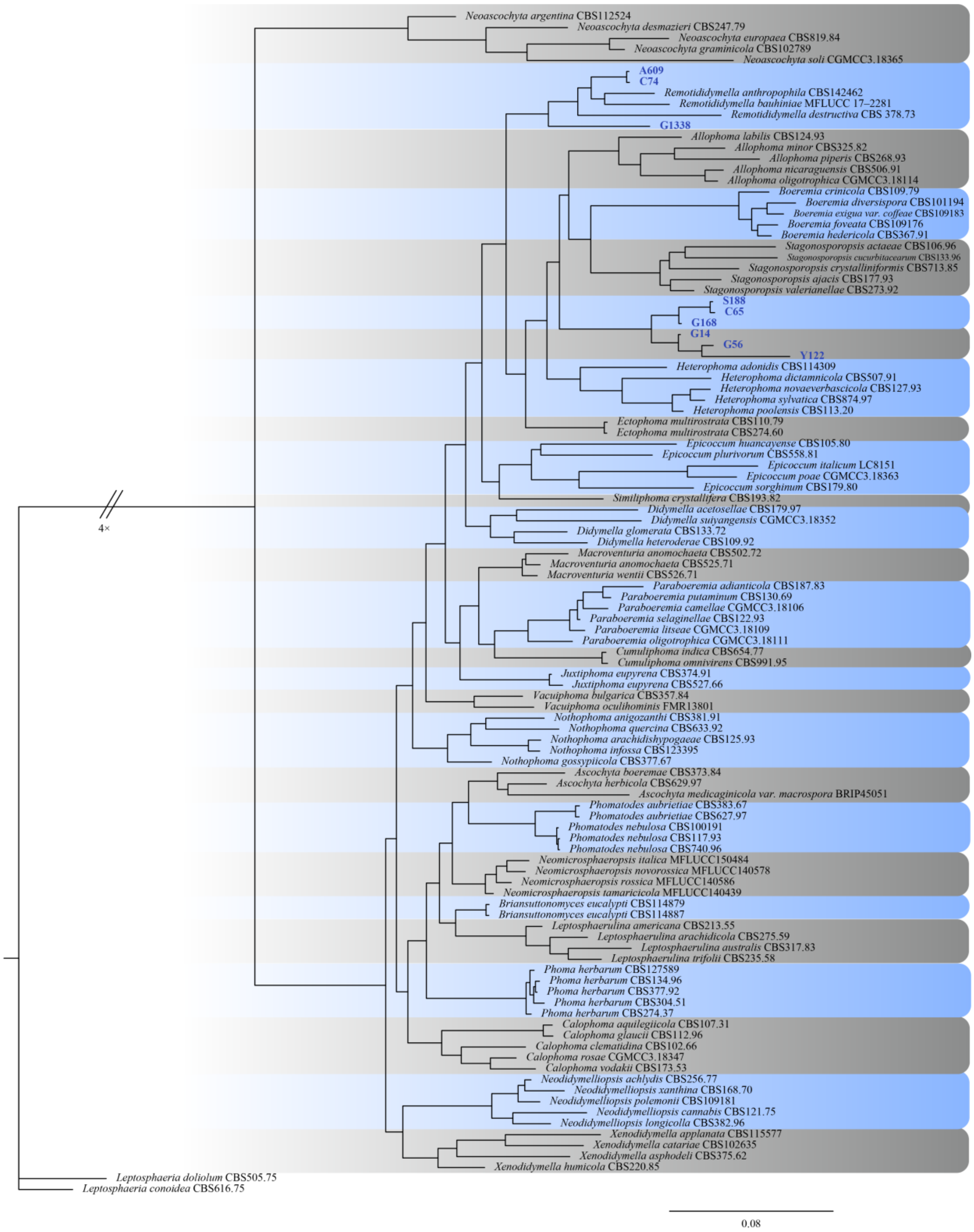

Supplementary Figure 2. Phylogenetic tree inferred from a Bayesian analysis based on a concatenated alignment of LSU, ITS, *rpb2*, and *tub2* sequences of representative strains of all Didymellaceae species based on 25 known genera. To adapt the figure to the page, some branches were shortened (shown with a double slash) and marked its shortened multiple.

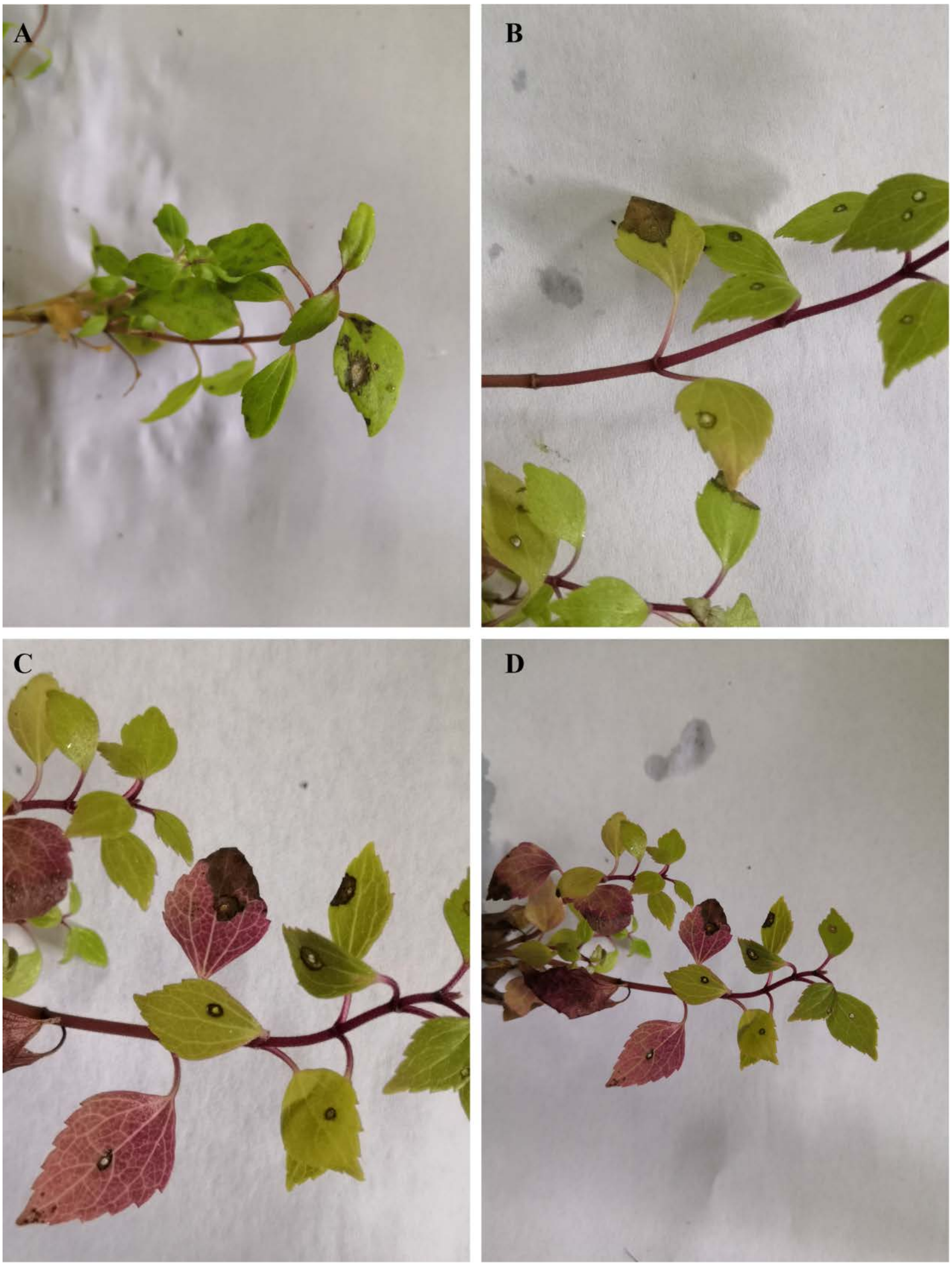

Supplementary Figure 3. The virulence of Y122 (A and B) and S188 (C and D) against *A. adenophora* with spray experiment.

2. Supplementary Tables

Supplementary Table 1. Information of the sampling sites.

Sampling sites		Longitude (E, °)	Latitude (N, °)	Elevation (m)
Invaded ranges	CY	99.54	23.28	1760
	ES	102.35	24.15	1590
	KM	102.62	24.98	2080
	NE	101.08	23.02	1430
	LC	100.04	22.62	1250
	SM	100.81	22.75	1320
	WS	100.05	25.32	1728
	XM	99.76	22.62	1650
	NJ	100.30	24.87	1730
	MJ	101.69	23.52	1560
	YL	99.44	25.88	2128
	YX	100.23	24.66	1580
Non-invaded ranges	CB	104.84	27.56	1720
	JC	99.95	26.45	2310
	LP	99.4	26.41	2280
	XCB	104.18	27.74	920
	YL	104.14	27.69	1760
	ZX	104.81	27.42	1510

Supplementary Table 2. The reference strains used in this study and their GenBank accession numbers.

Species	Strain number	Host, substrate	Country	GenBank accession numbers			
				LSU	ITS	rpb2	tub2
Allophoma labilis	CBS 124.93; PD 87/269	*Lycopersicon esculentum*	Netherlands	GU238091	GU237765	KT389552	GU237619
Al. minor	CBS 325.82	*Syzygium aromaticum*	Indonesia	GU238107	GU237831	KT389553	GU237632
Al. nicaraguensis	CBS 506.91; PD 91/876; IMI 215229	*Coffea arabica*	Nicaragua	GU238058	GU237876	KT389551	GU237596
Al. oligotrophica	CGMCC 3.18114; LC 6245	Air	China	KY742194	KY742040	KY742128	KY742282
	CGMCC 3.18115; LC 6246	Air	China	KY742195	KY742041	KY742129	KY742283
	CGMCC 3.18115; LC 6246	Air	China	KY742196	KY742042	KY742130	KY742284
Al. piperis	CBS 268.93; CBS 108.93; PD 88/720	*Peperomia pereskiifolia*	Netherlands	GU238129	GU237816	KT389554	GU237644
	CBS 108.93; PD 90/2011	*Peperomia* sp.	Netherlands	GU238130	GU237921	KT389555	GU237645
Al. tropica	CBS 436.75; DSM 63365	*Saintpaulia ionantha*	Germany	GU238149	GU237864	KT389556	GU237663
Al. zantedeschiae	CBS 131.93; PD 69/140	*Calla* sp.	Netherlands	GU238159	FJ427084	KT389557	FJ427188
	CBS 229.32	*Cicer arietinum*	Romania	KT389690	KT389473	KT389558	KT389767
	ICMP 16850	*Lycopersicon esculentum*	Hungary	KY742197	KY742043	KY742131	KY742285
Ascochyta boeremae	CBS 372.84; PD 80/1246	*Pisum sativum*	Australia	KT389697	KT389480	—	KT389774
As. boeremae	CBS 373.84; PD 80/1247	*Pisum sativum*	Australia	KT389698	KT389481	KT389560	KT389775
As. fabae	CBS 524.77	*Phaseolus vulgaris*	Belgium	GU237963	GU237880	—	GU237526
	CBS 649.71	*Vicia faba*	Netherlands	GU237964	GU237902	—	GU237527
	PD 83/492	*Phaseolus vulgaris*	Netherlands	GU237965	GU237917	—	GU237528

Supplementary table 2. (Continued).

Species	Strain number	Host, substrate	Country	GenBank accession numbers			
				LSU	ITS	rpb2	tub2
As. lentis	CBS 370.84; PD 81/783	*Lens culinaris*	—	KT38969	KT38947	—	KT38976
As. medicaginicola var. macrospora	BRIP 45051; LC 5258	*Medicago sativa*	Australia	KY74219	KY74204	KY74213	KY74228
	CBS 112.53	*Medicago sativa*	USA	GU23810	GU23774	—	GU23762
	CBS 404.65; IMI 116999	*Medicago sativa*	Canada	GU23810	GU23785	KP330423	GU23762
As. medicaginicola var.	CBS 316.90	*Medicago sativa*	Czech	GU23810	GU23782	—	GU23763
As. nigripycnidia	CBS 116.96; PD 95/7930	*Vicia cracca*	Russia	GU23811	GU23775	—	GU23763
As. phacae	CBS 184.55	*Phaca alpina*	Switzerland	KT38969	KT38947	—	KT38976
As. pisi	CBS 122750; ATCC	*Pisum sativum*	USA	KT38969	KT38947	—	KT38977
	CBS 122751; ATCC	*Pisum sativum*	Canada	KP330444	KP330432	EU87486	KP330388
	CBS 122785; PD 78/517	*Pisum sativum*	Netherlands	GU23796	GU23776	—	GU23753
	CBS 126.54	*Pisum sativum*	Netherlands	EU75413	GU23777	DQ67796	GU23753
	CBS 108.49	*Juglans regia*	Netherlands	KT38969	KT38947	—	KT38977
As. premilcurensis	MFLUCC 14-0518	*Heracleum*	Italy	KT32669	KT32669	—	—
As. rabiei	CBS 206.30	—	—	KT38969	KT38947	KT38955	KT38977
	CBS 237.37	*Cicer arietinum*	Bulgaria	KT38969	KT38947	—	KT38977
	CBS 534.65	*Cicer arietinum*	India	GU23797	GU23788	KP330405	GU23753
As. syringae	CBS 545.72	*Syringa vulgaris*	Netherlands	KT38970	KT38948	—	KT38977

Supplementary table 2. (Continued).

Species	Strain number	Host, substrate	Country	GenBank accession numbers			
				LSU	ITS	rpb2	tub2
As. versabilis	CBS 876.97; PD 82/1008	*Silene* sp.	Netherlands	GU23815	GU23790	KT38956	GU23766
As. viciae	CBS 451.68	*Vicia sepium*	Netherlands	KT389701	KT389484	KT38956	KT389778
As. viciae-pannonicae	CBS 254.92	*Vicia pannonica*	Czech	KT389702	KT389485	—	KT389779
Boeremia crinicola	CBS 109.79; PD 77/747	*Crinum powellii*	Netherlands	GU23792	GU23773	KT38956	GU23748
B. diversispora	CBS 102.80; IMI 331907; PD 79/61	*Phaseolus vulgaris*	Kenya	GU23793	GU23772	KT38956	GU23749
	CBS 101194; PD 79/687; IMI 373349	*Phaseolus vulgaris*	Netherlands	GU23792	GU23771	KT38956	GU23749
B. exigua var. coffeae	CBS 119730	*Coffea arabica*	Brazil	GU23794	GU23775	KT38956	GU23750
	CBS 109183; PD 2000/10506	*Coffea arabica*	Cameroon	GU23794	GU23774	KT38956	GU23750
	CBS 431.74; PD 74/2447	*Solanum tuberosum*	Netherlands	EU754183	FJ427001	KT38956	FJ427112
B. exigua var. forsythiae	CBS 101197; PD 95/721	*Forsythia* sp.	Netherlands	GU23793	GU23771	KT38957	GU23749
	CBS 101213; PD 92/959	*Forsythia* sp.	Netherlands	GU23793	GU23772	KT38957	GU23749
B. exigua var. gilvescens	CBS 101150; PD 79/118	*Cichorium intybus*	Netherlands	EU754182	GU23771	KT38956	GU23749
B. exigua var.	CBS 443.94	*Nerium oleander*	Italy	GU23793	GU23786	KT38957	GU23749
	CBS 101196; PD 79/176	*Nerium oleander*	France	GU23793	GU23771	KT38957	GU23749
B. exigua var. linicola	CBS 114.28	*Linum*	Netherlands	GU23793	GU23775	—	GU23749
	CBS 116.76; ATCC 32332; IMI	*Linum*	Netherlands	GU23793	GU23775	KT38957	GU23750
	CBS 248.38	*Nemophila insignis*	Netherlands	KT389703	KT389486	KT38957	KT389780

Supplementary table 2. (Continued).

Species	Strain number	Host, substrate	Country	GenBank accession numbers			
				LSU	ITS	rpb2	tub2
B. exigua var. opuli	CGMCC 3.18354; LC 8117	*Viburnum opulus*	USA	KY74219	KY74204	KY74213	KY74228
	LC 8118	*Viburnum opulus*	USA	KY74220	KY74204	KY74213	KY74228
B. exigua var. populi	CBS 100167; PD 93/217	*Populus* (×)	Netherland	GU23793	GU23770	—	GU23750
B. exigua var.	CBS 423.67	*Lathyrus* sp.	Netherland	KT389704	KT389487	KT389576	KT389781
	CBS 462.67	*Lamium maculatum*	Netherland	KT389705	KT389488	—	KT389782
	CBS 101207; PD 94/614	*Syringa vulgaris*	Netherland	GU23794	GU23772	—	GU23750
B. exigua var. viburni	CBS 100354; PD 83/448	*Viburnum opulus*	Netherland	GU23794	GU23771	KT389577	GU23750
B. foveata	CBS 109176; PD 94/1394	*Solanum tuberosum*	Bulgaria	GU23794	GU23774	KT389578	GU23750
B. hedericola	CBS 367.91; PD 87/229	*Hedera helix*	Netherland	GU23794	GU23784	KT389579	GU23751
B. lilacis	CBS 569.79; PD 72/741; IMI 331909	*Syringa vulgaris*	Netherland	GU23793	GU23789	—	GU23749
	CBS 588.67	*Philadelphus* sp.	Netherland	KT389709	KT389492	—	KT389786
	LC 5178	*Lonicera japonica*	China	KY74220	KY74204	—	KY74228
	LC 8116	*Ocimum* sp.	China	KY74220	KY74204	—	KY74229
B. lycopersici	CBS 378.67; PD 67/276	*Lycopersicon esculentum*	Netherland	GU23795	GU23784	KT389580	GU23751
B. noackiana	CBS 100353; PD 87/718	*Phaseolus vulgaris*	Guatemala	GU23795	GU23771	—	GU23751
	CBS 101203; PD 79/1114	*Phaseolus vulgaris*	Colombia	GU23795	GU23772	KT389581	GU23751
B. sambuci-nigrae	CBS 629.68; CECT 20048; IMI	*Sambucus nigra*	Netherland	GU23795	GU23789	—	GU23751

Supplementary table 2. (Continued).

Species	Strain number	Host, substrate	Country	GenBank accession numbers			
				LSU	ITS	rpb2	tub2
B. strasseri	CBS 126.93; PD 73/642	*Mentha* sp.	Netherlands	GU237956	GU237773	KT389584	GU237518
B. telephii	CBS 760.73; PD 71/1616	*Sedum telephium*	Netherlands	GU237959	GU237905	—	GU237521
	CBS 109175; PD 79/524	*Sedum telephium*	Netherlands	GU237958	GU237741	KT389585	GU237520
B. trachelospermi	CGMCC 3.18222; LC 8105	*Trachelospermum jasminoides*	USA	KY064032	KY064028	KY064033	KY064051
Briansuttonomyces eucalypti	CBS 114879; CPC 362	*Eucalyptus* sp.	South Africa	KU728519	KU728479	—	KU728595
	CBS 114887; CPC 363	*Eucalyptus* sp.	South Africa	KU728520	KU728480	—	KU728596
Calophoma aquilegiicola	CBS 107.31	*Aquilegia* sp.	—	KT389710	KT389493	—	KT389787
C. aquilegiicola	CBS 107.96; PD 73/598	*Aconitum pyramidale*	Netherlands	GU238041	GU237735	KT389586	GU237581
	CBS 108.96; PD 79/611	*Aquilegia* sp.	Netherlands	GU238042	GU237736	—	GU237582
	CBS 109.96; PD 83/832	*Aquilegia* sp.	Netherlands	KT389711	KT389494	—	KT389788
	CBS 116402	*Thalictrum dipterocarpum*	New Zealand	KT389712	KT389495	—	KT389789
C. clematidina	CBS 102.66	*Clematis* sp.	UK	FJ515630	FJ426988	KT389587	FJ427099
	CBS 108.79; PD 78/522	*Clematis* sp.	Netherlands	FJ515632	FJ426989	KT389588	FJ427100
C. clematidis-rectae	CBS 507.63; PD 07/03486747	*Clematis* sp.	Netherlands	FJ515647	FJ515606	KT389589	FJ515624
C. glaucii	CBS 112.96; PD 79/765	*Dicentra* sp.	Netherlands	GU238077	GU237750	—	GU237610
	CBS 114.96; PD 94/888	*Chelidonium majus*	Netherlands	FJ515649	FJ515609	—	FJ515627
C. rosae	CGMCC 3.18347; LC 5169	*Rosa* sp.	China	KY742203	KY742049	KY742135	KY742291

Supplementary table 2. (Continued).

Species	Strain number	Host, substrate	Country	GenBank accession numbers			
				LSU	ITS	rpb2	tub2
	LC 8119	*Rosa* sp.	China	KY742204	KY742050	KY742136	KY742292
C. vodakii	CBS 173.53	*Hepatica triloba*	Switzerland	KT389714	KT389497	—	KT389791
Cumuliphoma indica	CBS 654.77； FMR 15341	Unknown	India	GU238122	FJ427043	LT623261	FJ427153
	CBS 991.95； FMR 15331	Soil	Papua New Guinea	GU238121	FJ427044	LT623262	FJ427154
Didymella acetosellae	CBS 179.97	*Rumex* hydrolapathum	Netherlands	GU238034	GU237793	KP330415	GU237575
D. aeria	CGMCC 3.18353; LC 7441	Air	China	KY742205	KY742051	KY742137	KY742293
	LC 8120	Air	China	KY742206	KY742052	KY742138	KY742294
D. aliena	CBS 379.93; PD 82/945	*Berberis* sp.	Netherlands	GU238037	GU237851	KP330416	GU237578
	LC 8121	*Pyrus calleryana*	Italy	KY742207	KY742053	—	KY742295
D. americana	CBS 185.85; PD 80/1191	*Zea mays*	USA	GU237990	FJ426972	KT389594	FJ427088
	CBS 568.97; ATCC 44494;	*Glycine max*	USA	GU237991	FJ426974	—	FJ427090
	LC 5157	*Sorghum bicolor*	China	KY742208	KY742054	KY742139	KY742296
D. anserina	CBS 253.80	—	Germany	KT389715	KT389498	KT389595	KT389795
	CBS 285.29	*Calluna* sp.	UK	KT389716	KT389499	—	KT389796
	CBS 360.84	*Potato flour*	Netherlands	GU237993	GU237839	KT389596	GU237551
	CBS 397.65	Plastic	Germany	KT389717	KT389500	KT389597	KT389797
D. aquatica	CGMCC 3.18349; LC 5556	Water	China	KY742209	KY742055	KY742140	KY742297

Supplementary table 2. (Continued).

Species	Strain number	Host, substrate	Country	GenBank accession numbers			
				LSU	ITS	rpb2	tub2
	LC 5555	Water	China	KY742210	KY742056	KY742141	KY742298
D. arachidicola	CBS 333.75; ATCC 28333	*Arachis hypogaea*	South Africa	GU237996	GU237833	KT389598	GU237554
D. aurea	CBS 269.93; PD 78/1087	*Medicago polymorpha*	New Zealand	GU237999	GU237818	KT389599	GU237557
D. bellidis	CBS 714.85; PD 74/265	*Bellis perennis*	Netherlands	GU238046	GU237904	KP330417	GU237586
	PD 94/886	*Bellis* sp.	Netherlands	GU238047	GU237923	—	GU237587
D. boeremae	CBS 109942; PD 84/402	*Medicago littoralis cv. Harbinger Harbinger*	Australia	GU238048	FJ426982	KT389600	FJ427097
D. calidophila	CBS 448.83	Soil	Egypt	GU238052	FJ427059	—	FJ427168
	PD 84/109	*Cucumis sativus*	Netherlands	GU238053	FJ427060	—	FJ427169
D. chenopodii	CBS 128.93; PD 79/140	*Chenopodium quinoa cv. Sajana Sajana*	Peru	GU238055	GU237775	KT389602	GU237591
D. chloroguttulata	CGMCC 3.18351; LC 7435	Air	China	KY742211	KY742057	KY742142	KY742299
	LC 8122	Air	China	KY742212	KY742058	KY742143	KY742300
D. coffeae-arabicae	CBS 123380; PD 84/1013	*Coffea arabica*	Ethiopia	GU238005	FJ426993	KT389603	FJ427104
	LC 8975	*Lagerstroemia indica*	Italy	KY742213	KY742059	KY742144	KY742301
D. curtisii	CBS 251.92; PD 86/1145	*Nerine* sp.	Netherlands	GU238013	FJ427038	—	FJ427148
	PD 92/1460	*Sprekelia* sp.	Netherlands	GU238012	FJ427041	KT389604	FJ427151
D. dactylidis	CBS 124513; PD 73/1414	*Dactylis glomerata*	USA	GU238061	GU237766	—	GU237599
D. dimorpha	CBS 346.82	*Opuntia* sp	Spain	GU238068	GU237835	—	GU237606

Supplementary table 2. (Continued).

Species	Strain number	Host, substrate	Country	GenBank accession numbers			
				LSU	ITS	rpb2	tub2
D. ellipsoidea	CGMCC 3.18350; LC 7434	Air	China	KY742214	KY742060	KY742145	KY742302
	LC 8123	Air	China	KY742215	KY742061	KY742146	KY742303
D. eucalyptica	CBS 377.91; PD 79/210	*Eucalyptus* sp.	Australia	GU238007	GU237846	KT389605	GU237562
D. exigua	CBS 183.55	*Rumex arifolius*	France	EU754155	GU237794	EU874850	GU237525
D. gardeniae	CBS 626.68; IMI 108771	*Gardenia jasminoides*	India	GQ387595	FJ427003	KT389606	FJ427114
D. glomerata	CBS 133.72	*Fresco in church*	Romania	KT389718	FJ427004	—	FJ427115
	CBS 528.66; PD 63/590	*Chrysanthemum* sp.	Netherlands	EU754184	FJ427013	GU371781	FJ427124
	LC 4963	*Leymus chinensis*	China	KY742216	KY742062	KY742147	KY742304
	LC 8124	*Faeces*	China	KY742217	KY742063	KY742148	KY742305
D. heteroderae	CBS 109.92; PD 73/1405	Undefined food material	Netherlands	GU238002	FJ426983	KT389601	FJ427098
	LC 8125	*Hydrangea macrophylla*	China	KY742218	KY742064	KY742149	KY742306
D. ilicicola	CGMCC 3.18355; LC 8126	*Ilex chinensis*	Italy	KY742219	KY742065	KY742150	KY742307
	LC 8127	*Ilex chinensis*	Italy	KY742220	KY742066	KY742151	KY742308
D. infuscatispora	CGMCC 3.18356; LC 8128	*Chrysanthemum indicum*	China	KY742221	KY742067	KY742152	KY742309
	LC 8129	*Chrysanthemum indicum*	China	KY742222	KY742068	—	KY742310
D. lethalis	CBS 103.25	—	—	GU238010	GU237729	KT389607	GU237564
	LC 8130	*Liquidambar styraciflua*	Italy	KY742223	KY742069	KY742153	KY742311

Supplementary table 2. (Continued).

Species	Strain number	Host, substrate	Country	GenBank accession numbers			
				**LSU**	**ITS**	**rpb2**	**tub2**
D. longicolla	CBS 124514; PD 80/1189	*Opuntia* sp.	Spain	GU238095	GU237767	—	GU237622
D. macrophylla	CGMCC 3.18357; LC 8131	*Hydrangea macrophylla*	Italy	KY742224	KY742070	KY742154	KY742312
	LC 8132	*Hydrangea macrophylla*	Italy	KY742225	KY742071	KY742155	KY742313
D. mascrostoma	CBS 223.69	*Acer pseudoplatanus*	Switzerland	GU238096	GU237801	KT389608	GU237623
	CBS 247.38	*Pinus nigra var. astriaca*	—	KT389719	KT389501	—	KT389798
	CBS 482.95	*Larix decidua*	Germany	GU238099	GU237869	KT389609	GU237626
	CBS 529.66; PD 66/521	*Malus sylvestris*	Netherlands	GU238098	GU237885	—	GU237625
	LC 5203	Soil	China	KY742226	KY742072	KY742156	KY742314
D. maydis	CBS 588.69	*Zea mays*	USA	EU754192	FJ427086	GU371782	FJ427190
D. microchlamydospora	CBS 105.95	*Eucalyptus* sp.	UK	GU238104	FJ427028	KP330424	FJ427138
D. molleriana	CBS 229.79; LEV 7660	*Digitalis purpurea*		GU238067	GU237802	KP330418	GU237605
	CBS 109179; PD 90/835-1	*Digitalis* sp.	Netherlands	GU238066	GU237744	—	GU237604
D. musae	CBS 463.69	*Mangifera indica*	India	GU238011	FJ427026	—	FJ427136
D. negriana	CBS 358.71	*Vitis vinifera*	Germany	GU238116	GU237838	KT389610	GU237635
	ICMP 10845; LC 5249	*Vitis vinifera*	former Yugoslavia	KY742227	KY742073	—	KY742315
D. nigricans	CBS 444.81; PDDCC 6546	*Actinidia chinensis*	New Zealand	GU238000	GU237867	—	GU237558
	LC 8133	*Robinia pseudoacacia f. decaisneana decaisneana*	Italy	KY742228	KY742074	KY742157	KY742316

Supplementary table 2. (Continued).

Species	Strain number	Host, substrate	Country	GenBank accession numbers			
				LSU	ITS	rpb2	tub2
	LC 8134	*Acer palmatum*	Japan	KY74222	KY74207	KY74215	KY74231
	LC 8135	*Acer palmatum*	Japan	KY74223	KY74207	KY74215	KY74231
	LC 8136	*Acer palmatum*	Japan	KY74223	KY74207	KY74216	KY74231
	PD 77/919	*Actinidia chinensis*	New	GU23800	GU23791	KT38961	GU23755
D. ocimicola	CGMCC 3.18358; LC 8137	*Ocimum* sp.	China	KY74223	KY74207	—	KY74232
	LC 8138	*Ocimum* sp.	China	KY74223	KY74207	—	KY74232
D. pedeiae	CBS 124517; PD 92/612A	*Schefflera*	Netherlands	GU23812	GU23777	KT38961	GU23764
D. pinodella	CBS 318.90; PD 81/729	*Pisum sativum*	Netherlands	GU23801	FJ427051	—	FJ427161
	CBS 531.66	*Trifolium pretense*	USA	GU23801	FJ427052	KT38961	FJ427162
	LC 8139	*Acer palmatum*	Japan	KY74223	KY74208	KY74216	KY74232
D. pinodes	CBS 525.77	*Pisum sativum*	Belgium	GU23802	GU23788	KT38961	GU23757
D. pomorum	CBS 285.76; ATCC 26241; IMI	*Heracleum dissectum*	Russia	GU23802	FJ427053	KT38961	FJ427163
	CBS 354.52	*Triticum spelta*	Switzerland	KT38972	KT38950	KT38961	KT38979
	CBS 388.80	*Triticum* sp.	South Africa	GU23802	FJ427055	KT38961	FJ427165
	CBS 539.66; ATCC 16791; IMI	*Polygonum tataricum*	Netherlands	GU23802	FJ427056	KT38961	FJ427166
	LC 5185	*Gentiana straminea*	China	KY74223	KY74208	KY74216	KY74232
	LC 8140	*Dendrobium*	China	KY74223	KY74208	—	KY74232

Supplementary table 2. (Continued).

Species	Strain number	Host, substrate	Country	GenBank accession numbers			
				LSU	ITS	rpb2	tub2
D. protuberans	CBS 132.96; PD 93/853	*Rhinanthus major*	Netherlands	GU237989	GU237778	—	GU237550
	CBS 377.93; PD 80/976	*Daucus carota*	Netherlands	GU238014	GU237847	KT389619	GU237565
	CBS 381.96; PD 71/706	*Lycium halifolium*	Netherlands	GU238029	GU237853	KT389620	GU237574
	CBS 391.93; PD 80/87	*Spinacia oleracea*	Netherlands	GU238015	GU237858	KT389621	GU237566
D. pteridis	CBS 379.96	*Pteris* sp.	Netherlands	KT389722	KT389504	KT389624	KT389801
D. rhei	BRIP 5562; LC 5251	*Rheum rhaponticum*	Australia	KY742237	KY742083	KY742163	KY742325
	CBS 109177; LEV 15165; PD 2000/9941	*Rheum rhaponticum*	New Zealand	GU238139	GU237743	KP330428	GU237653
D. rumicicola	CBS 683.79; LEV 15094	*Rumex obtusifolius*	New Zealand	KT389721	KT389503	KT389622	KT389800
D. sancta	CBS 281.83	*Ailanthus altissima*	South Africa	GU238030	FJ427063	KT389623	FJ427170
D. segeticola	CGMCC 3.17489; LC 1636	*Cirsium segetum*	China	KP330455	KP330443	KP330414	KP330399
	CGMCC 3.17498; LC 1635	*Cirsium segetum*	China	KP330454	KP330442	KP330413	KP330398
	LC 1633	*Cirsium segetum*	China	KP330452	KP330440	KP330411	KP330396
	LC 1634	*Cirsium segetum*	China	KP330453	KP330441	KP330412	KP330397
	LC 8141	*Camellia sasanqua*	Japan	KY742238	KY742084	KY742164	KY742326
D. senecionicola	CBS 160.78; LEV 11451	*Senecio jacobaea*	New Zealand	GU238143	GU237787	—	GU237657
D. sinensis	CGMCC 3.18348; LC 5210	*Cerasus pseudocerasus*	China	KY742239	KY742085	—	KY742327
	LC 5246	Urticaceae	China	KY742240	KY742086	KY742165	KY742328

Supplementary table 2. (Continued).

Species	Strain number	Host, substrate	Country	GenBank accession numbers			
				LSU	ITS	rpb2	tub2
	LC 8142	*Dendrobium*	China	KY74224	KY74208	KY74216	KY74232
	LC 8143	*Dendrobium*	China	KY74224	KY74208	KY74216	KY74233
D. subglomerata	CBS 110.92; PD 76/1010	*Triticum* sp.	USA	GU23803	FJ427080	KT38962	FJ427186
D. subherbarum	CBS 249.92; PD 78/1088	*Solanum* sp.	Peru	GU23814	GU23780	—	GU23765
	CBS 250.92; DAOM 171914; PD	*Zea mays*	Canada	GU23814	GU23780	—	GU23765
D. suiyangensis	CGMCC 3.18352; LC 7439	Air	China	KY74224	KY74208	KY74216	KY74233
	LC 8144	Air	China	KY74224	KY74209	KY74216	KY74233
D. viburnicola	CBS 523.73; PD 69/800	*Viburnum cassioides*	Netherlands	GU23815	GU23787	KP33043	GU23766
Ectophoma multirostrata	CBS 110.79；FMR 15342	*Cucumis sativus*	The	GU23811	FJ427030	LT62326	FJ427140
	CBS 274.60；FMR 15335	Soil	Maharashtra	GU23811	FJ427031	LT62326	FJ427141
Epicoccum brasiliense	CBS 120105	*Amaranthus* sp.	Brazil	GU23804	GU23776	KT38962	GU23758
E. camelliae	CGMCC 3.18343; LC 4858	*Camellia sinensis*	China	KY74224	KY74209	KY74217	KY74233
	LC 4862	*Camellia sinensis*	China	KY74224	KY74209	KY74217	KY74233
E. dendrobii	CGMCC 3.18359; LC 8145	*Dendrobium*	China	KY74224	KY74209	—	KY74233
	LC 8146	*Dendrobium*	China	KY74224	KY74209	—	KY74233
E. draconis	CBS 186.83; PD 82/47	*Dracaena* sp.	Rwanda	GU23807	GU23779	KT38962	GU23760
E. duchesneae	CGMCC 3.18345; LC 5139	*Duchesnea indica*	China	KY74224	KY74209	—	KY74233

Supplementary table 2. (Continued).

Species	Strain number	Host, substrate	Country	GenBank accession numbers			
				LSU	ITS	rpb2	tub2
	LC 8147	*Duchesnea indica*	China	KY742250	KY742096	—	KY742338
E. henningsii	CBS 104.80; PD 74/1017	*Acacia mearnsii*	Kenya	GU238081	GU237731	KT389629	GU237612
E. hordei	CGMCC 3.18360; LC 8148	*Hordeum vulgare*	Australia	KY742251	KY742097	—	KY742339
	LC 8149	*Hordeum vulgare*	Australia	KY742252	KY742098	—	KY742340
E. huancayense	CBS 105.80; PD 75/908	*Solanum* sp.	Peru	GU238084	GU237732	KT389630	GU237615
E. italicum	CGMCC 3.18361; LC 8150	*Acca sellowiana*	Italy	KY742253	KY742099	KY742172	KY742341
	LC 8151	*Acca sellowiana*	Italy	KY742254	KY742100	KY742173	KY742342
E. latusicollum	CGMCC 3.18346; LC 5158	*Sorghum bicolor*	China	KY742255	KY742101	KY742174	KY742343
	LC 4859	*Camellia sinensis*	China	KY742256	KY742102	KY742175	KY742344
	LC 5124	*Vitex negundo*	China	KY742257	KY742103	—	KY742345
	LC 8152	*Podocarpus macrophyllus*	Japan	KY742258	KY742104	KY742176	KY742346
	LC 8153	*Podocarpus macrophyllus*	Japan	KY742259	KY742105	KY742177	KY742347
	LC 8154	*Acer palmatum*	Japan	KY742260	KY742106	—	KY742348
E. layuense	CGMCC 3.18362; LC 8155	*Perilla* sp.	China	KY742261	KY742107	—	KY742349
	LC 8156	*Perilla* sp.	China	KY742262	KY742108	—	KY742350
E. nigrum	CBS 125.82; IMI 331914; CECT 20044	*Human toenail*	Netherlands	GU237974	FJ426995	KT389631	FJ427106
	CBS 173.73; ATCC 24428; IMI 164070	*Dactylis glomerata*	USA	GU237975	FJ426996	KT389632	FJ427107

Supplementary table 2. (Continued).

Species	Strain number	Host, substrate	Country	GenBank accession numbers			
				LSU	ITS	rpb2	tub2
	LC 5180	*Lonicera japonica*	China	KY74226	KY74210	KY74217	KY74235
	LC 8157	*Ocimum* sp.	China	KY74226	KY74211	KY74217	KY74235
	LC 8158	*Poa annua*	USA	KY74226	KY742111	KY74218	KY74235
	LC 8159	*Poa annua*	USA	KY74226	KY74211	KY74218	KY74235
E. pimprinum	CBS 246.60; ATCC 22237; ATCC 16652	Soil	India	GU23797	FJ427049	—	FJ427159
	PD 77/1028	Soil	India	GU23797	FJ427050	KT389633	FJ427160
E. plurivorum	CBS 558.81; PDDCC 6873	*Setaria* sp.	New	GU23813	GU23788	KT389634	GU23764
E. poae	CGMCC 3.18363; LC 8160	*Poa annua*	USA	KY74226	KY74211	KY74218	KY74235
	LC 8161	*Poa annua*	USA	KY74226	KY74211	KY74218	KY74235
	LC 8162	*Poa annua*	USA	KY74226	KY74211	KY74218	KY74235
E. sorghinum	CBS 179.80; PD 76/1018	*Sorghum vulgare*	Puerto Rico	GU23797	FJ427067	KT389635	FJ427173
	CBS 627.68; PD 66/926	*Citrus* sp.	France	GU23797	FJ427072	KT389636	FJ427178
	LC 4860	*Camellia sinensis*	China	KY74227	KY74211	KY74218	KY74235
E. viticis	BRIP 29294; LC 5257	*Andropogon*	Australia	KY74227	KY74211	—	KY74235
	CGMCC 3.18344; LC 5126	*Vitex negundo*	China	KY74227	KY74211	KY74218	KY74236
Heterophoma	CBS 114309; UPSC 2982	*Adonis vernalis*	Sweden	KT389724	KT389506	KT389637	KT389803
H. dictamnicola	CBS 507.91; PD 74/148	*Dictamnus albus*	Netherlands	GU23806	GU23787	KT389638	GU23760

Supplementary table 2. (Continued).

Species	Strain number	Host, substrate	Country	GenBank accession numbers			
				LSU	ITS	rpb2	tub2
H. novae-verbascicola	CBS 127.93; PD 92/347	*Verbascum*	Netherlands	GU23812	GU23777	—	GU23763
H. poolensis	CBS 113.20; PD 92/774	—	—	GU23811	GU23775	—	GU23763
	CBS 116.93; PD 71/884	*Antirrhinum majus*	Netherlands	GU23813	GU23775	—	GU23764
H. sylvatica	CBS 874.97; PD 93/764	*Melampyrum*	Netherlands	GU23814	GU23790	—	GU23766
H. verbascicola	CGMCC 3.18364; LC 8163	*Verbascum thapsus*	China	KY74227	KY74211	KY74218	KY74236
	LC 8164	*Verbascum thapsus*	China	KY74227	KY74212	KY74218	KY74236
Juxtiphoma eupyrena	CBS 374.91； FMR 15329	*Solanum tuberosum*	The	GU23807	FJ426999	LT62326	FJ427110
	CBS 527.66； FMR 15337	Wheat field soil	Germany	GU23807	FJ427000	LT62326	FJ427111
Leptosphaeria conoidea	CBS 616.75; ATCC 32813; IMI 199777	*Lunaria annua*	Netherlands	JF740279	JF740201	KT38963	KT38980
Leptosphaeria doliolum	CBS 505.75	*Urtica dioica*	Netherlands	GQ38757	JF740205	KT38964	JF740144
Leptosphaerulina	CBS 213.55	*Trifolium pratense*	USA	GU23798	GU23779	KT38964	GU23753
L. arachidicola	CBS 275.59; ATCC 13446	*Arachis hypogaea*	Taiwan, China	GU23798	GU23782	—	GU23754
L. australis	CBS 317.83	*Eugenia aromatica*	Indonesia	EU75416	GU23782	GU37179	GU23754
L. trifolii	CBS 235.58	*Trifolium* sp.	Netherlands	GU23798	GU23780	—	GU23754
Macroventuria	CBS 502.72	*Medicago sativa*	South Africa	GU23798	GU23787	—	GU23754
	CBS 525.71	*Decayed canvas*	South Africa	GU23798	GU23788	GU45634	GU23754
Macroventuria wentii	CBS 526.71	Plant litter	USA	GU23798	GU23788	KT38964	GU23754

Supplementary table 2. (Continued).

Species	Strain number	Host, substrate	Country	GenBank accession numbers			
				LSU	ITS	rpb2	tub2
Neoascochyta argentina	CBS 112524	*Triticum aestivum*	Argentina	KT389742	KT389524	—	KT389822
Neoa. desmazieri	CBS 247.79	Poaceae	Austria	KT389725	KT389507	—	KT389805
	CBS 297.69	*Lolium perenne*	Germany	KT389726	KT389508	KT389644	KT389806
	CBS 758.97	Hay	Norway	KT389727	KT389509	—	KT389807
Neoa. europaea	CBS 819.84	*Hordeum vulgare*	Germany	KT389728	KT389510	KT389645	KT389808
	CBS 820.84	*Hordeum vulgare*	Germany	KT389729	KT389511	KT389646	KT389809
Neoa. exitialis	CBS 118.40	—	—	KT389732	KT389514	KT389647	KT389812
	CBS 389.86	*Triticum aestivum*	Switzerland	KT389733	KT389515	KT389648	KT389813
	CBS 811.84	*Secale cereale*	Germany	KT389734	KT389516	—	KT389814
	CBS 812.84	*Hordeum vulgare*	Germany	KT389735	KT389517	—	KT389815
	CBS 110124	*Triticum* sp.	Netherlands	KT389730	KT389512	—	KT389810
	CBS 113693; UPSC 1929	*Allium* sp.	Sweden	KT389731	KT389513	—	KT389811
Neoa. graminicola	CBS 301.69	*Lolium multiflorum*	Germany	KT389737	KT389519	KT389650	KT389817
	CBS 447.82	*Triticum aestivum*	Germany	KT389738	KT389520	—	KT389818
	CBS 586.79	*Hordeum vulgare*	Belgium	KT389739	KT389521	—	KT389819
	CBS 815.84	*Hordeum vulgare*	Germany	KT389740	KT389522	—	KT389820
	CBS 816.84	*Hordeum vulgare*	Germany	KT389741	KT389523	KT389651	KT389821

Supplementary table 2. (Continued).

Species	Strain number	Host, substrate	Country	GenBank accession numbers			
				LSU	ITS	rpb2	tub2
	CBS 102789	*Lolium perenne*	New Zealand	KT389736	KT389518	KT389649	KT389816
Neoa. paspali	CBS 560.81; PD 92/1569	*Paspalum dilatatum*	New Zealand	GU238124	FJ427048	KP330426	FJ427158
Neoa. soli	CGMCC 3.18365; LC 8165	Soil	China	KY742275	KY742121	—	KY742363
	LC 8166	Soil	China	KY742276	KY742122	—	KY742364
Neoa. triticicola	CBS 544.74	*Triticum aestivum*	South Africa	EU754134	GU237887	KT389652	GU237488
Neodidymelliopsis achlydis	CBS 256.77	*Achlys triphylla*	Canada	KT389749	KT389531	—	KT389829
Neod. cannabis	CBS 121.75; ATCC 32164	*Urtica dioica*	Netherlands	GU237972	GU237761	—	GU237535
	CBS 234.37	*Cannabis sativa*	—	GU237961	GU237804	KP330403	GU237523
	CBS 591.67	*Urtica dioica*	Netherlands	KT389746	KT389528	—	KT389826
	CBS 629.76	*Packing material*	Netherlands	KT389747	KT389529	—	KT389827
Neod. longicolla	CBS 382.96	*Soil in desert*	Israel	KT389750	KT389532	—	KT389830
Neod. polemonii	CBS 375.67	*Polemonium caeruleum*	Netherlands	KT389748	KT389530	—	KT389828
	CBS 109181; PD 83/757	*Polemonium caeruleum*	Netherlands	GU238133	GU237746	KP330427	GU237648
Neod. xanthina	CBS 168.70	*Delphinium* sp.	Netherlands	KT389751	KT389533	—	KT389831
	CBS 383.68	*Delphinium* sp.	Netherlands	GU238157	GU237855	KP330431	GU237668
Neomicrosphaeropsis italica	MFLUCC 15-0485; ICMP 21253	*Tamarix* sp.	Italy	KU729854	KU900318	KU674820	—
	MFLUCC 15-0484	*Tamarix* sp.	Italy	KU729853	KU900319	KU695539	KX453298

Supplementary table 2. (Continued).

Species	Strain number	Host, substrate	Country	GenBank accession numbers			
				LSU	ITS	rpb2	tub2
	MFLUCC 16-0284	*Tamarix* sp.	Italy	KU900296	KU900321	KU714604	KX453299
Neom. novorossica	MFLUCC 14-0578	*Tamarix ramosissima*	Russia	KX198710	KX198709	—	—
Neom. rossica	MFLUCC 14-0586	*Tamarix ramosissima*	Russia	KU729855	KU752192	—	—
Neom. tamaricicola	MFLUCC 14-0443	*Tamarix gallica*	Italy	KU729851	KU900322	—	—
	MFLUCC 14-0439	*Tamarix gallica*	Italy	KU729858	KU900323	—	—
Nothophoma anigozanthi	CBS 381.91; PD 79/1110	*Anigozanthus maugleisii*	Netherlands	GU238039	GU237852	KT389655	GU237580
No. arachidis-hypogaeae	CBS 125.93; PD 77/1029	*Arachis hypogaea*	India	GU238043	GU237771	KT389656	GU237583
No. gossypiicola	CBS 377.67	*Gossypium* sp.	USA	GU238079	GU237845	KT389658	GU237611
No. infossa	CBS 123395	*Fraxinus pennsylvanica*	Argentina	GU238089	FJ427025	KT389659	FJ427135
No. quercina	CBS 633.92; ATCC 36786	Microsphaera alphitoides from *Quercus* sp.	Ukraine	EU754127	GU237900	KT389657	GU237609
Paraboeremia adianticola	CBS 187.83; PD 82/128	*Polystichum adiantiforme*	USA	GU238035	GU237796	KP330401	GU237576
	CBS 260.92; PD 86/1103	*Pteris ensiformis*	—	KT389752	KT389534	—	KT389832
Pa. camellae	CGMCC 3.18106; LC 4852	*Camellia* sp.	China	KX829042	KX829034	KX829050	KX829058
	CGMCC 3.18107; LC 6253	*Camellia* sp.	China	KX829043	KX829035	KX829051	KX829059
	CGMCC 3.18108; LC 6254	*Camellia* sp.	China	KX829044	KX829036	KX829052	KX829060
Pa. litseae	CGMCC 3.18109; LC 5028	*Litsea* sp.	China	KX829037	KX829029	KX829045	KX829053
	CGMCC 3.18110; LC 5030	*Litsea* sp.	China	KX829038	KX829030	KX829046	KX829054

Supplementary table 2. (Continued).

Species	Strain number	Host, substrate	Country	GenBank accession numbers			
				LSU	ITS	rpb2	tub2
Pa. oligotrophica	CGMCC 3.18111; LC 6250	Carbonatite	China	KX82903	KX82903	KX829047	KX8290
	CGMCC 3.18112; LC 6251	Carbonatite	China	KX82904	KX82903	KX829048	KX8290
	CGMCC 3.18113; LC 6252	Carbonatite	China	KX82904	KX82903	KX829049	KX8290
Pa. putaminum	CBS 130.69; CECT 20054; IMI	*Malus sylvestris*	Denmark	GU23813	GU23777	—	GU2376
	CBS 372.91; PD 75/960	*Ulmus* sp.	Netherland	GU23813	GU23784	—	GU2376
Pa. selaginellae	CBS 122.93; PD 77/1049	*Selaginella* sp.	Netherland	GU23814	GU23776	—	GU2376
Phoma herbarum	CBS 134.96; PD 84/676	*Delphinium* sp.	Netherland	KT389753	KT389535	KT389661	KT3898
	CBS 274.37	*Picea excelsa*	UK	KT389754	KT389537	KT389662	KT3898
	CBS 304.51	*Achillea millefolium*	Switzerlan	KT389755	KT389538	—	KT3898
	CBS 377.92; IMI 213845	Human leg	Netherland	KT389756	KT389536	KT389663	KT3898
	CBS 502.91; PD 82/276	*Nerium* sp.	Netherland	GU23808	GU23787	KP330419	GU2376
	CBS 615.75; PD 73/665; IMI 199779	*Rosa multiflora cv.*	Netherland	EU754186	FJ427022	KP330420	FJ42713
	CBS 127589; UAMH 10909	*Polytrichum juniperinum*	USA	KT389757	KT389539	KT389664	KT3898
Phomatodes aubrietiae	CBS 383.67; PD 65/223	*Aubrietia hybrida cv. Superbissima*	Netherlands	GU23804 4	GU23785 4	—	GU2375 84
Phomat. aubrietiae	CBS 627.97; PD 70/714	*Aubrietia* sp.	Netherland	GU23804	GU23789	KT389665	GU2375
Phomat. nebulosa	CBS 117.93; PD 83/90	*Mercurialis perennis*	Netherland	GU23811	GU23775	KP330425	GU2376
	CBS 740.96	*Armoracia rusticana*	Netherland	KT389758	KT389540	KT389667	KT3898

Supplementary table 2. (Continued).

Species	Strain number	Host, substrate	Country	GenBank accession numbers			
				LSU	ITS	rpb2	tub2
	CBS 100191	*Thlaspi arvense*	Poland	KP330446	KP330434	KT389666	KP330390
Pseudohendersonia galiorum	MFLUCC 14–0452	*Galium* sp.	Italy	KU84820	—	—	—
Remotididymella	CBS 142462; UTHSC:DI16-278	*Human respiratory tract*	USA	LN907421	LT592936	LT593075	LT593005
R. destructiva	CBS 378.73; FMR 15328	*Lycopersicon esculentum*	Tonga	GU23806	GU23784	LT623258	GU23760
R. bauhiniae	MFLUCC 17–2281	*Bauhinia* sp.	Thailand	MK34795	MK34773	MK43491	MK41288
Similiphoma crystallifera	CBS 193.82; FMR 15343	*Chamaespartium*	Austria	GU23806	GU23779	LT623267	GU23759
Stagonosporopsis actaeae	CBS 106.96; PD 94/1318	*Actaea spicata*	Netherland	GU23816	GU23773	KT389672	GU23767
	CBS 114303; UPSC 2962	*Actaea spicata*	Sweden	KT389760	KT389544	—	KT389847
S. ajacis	CBS 177.93; PD 90/115	*Delphinium* sp.	Kenya	GU23816	GU23779	KT389673	GU23767
S. andigena	CBS 101.80; PD 75/909; IMI	*Solanum* sp.	Peru	GU23816	GU23771	—	GU23767
	CBS 269.80; PD 75/914	*Solanum* sp.	Peru	GU23817	GU23781	—	GU23767
S. artemisiicola	CBS 102636; PD 73/1409	*Artemisia dracunculus*	France	GU23817	GU23772	KT389674	GU23767
S. astragali	CBS 178.25; MUCL 9915	*Astragalus* sp.	—	GU23817	GU23779	—	GU23767
S. bomiensis	CGMCC 3.18366; LC 8167	*Boraginaceae*	China	KY74227	KY74212	KY74218	KY74236
	LC 8168	*Boraginaceae*	China	KY74227	KY74212	KY74219	KY74236
S. caricae	CBS 248.90	*Carica papaya*	Chile	GU23817	GU23780	—	GU23768
	CBS 282.76	*Brassica* sp.	Indonesia	GU23817	GU23782	—	GU23768

Supplementary table 2. (Continued).

Species	Strain number	Host, substrate	Country	GenBank accession numbers			
				LSU	ITS	rpb2	tub2
S. chrysanthemi	CBS 500.63; MUCL 8090	*Chrysanthemum indicum*	Germany	GU238190	GU237871	—	GU237695
	CBS 137.96; PD 84/75	*Chrysanthemum indicum*	Netherlands	GU238191	GU237783	—	GU237696
S. crystalliniformis	CBS 713.85; ATCC 76027; PD 83/826	*Lycopersicon esculentum*	Colombia	GU238178	GU237903	KT389675	GU237683
S. cucurbitacearum	CBS 133.96; PD 79/127	*Cucumis* sp.	New Zealand	GU238181	GU237780	KT389676	GU237686
S. dennisii	CBS 631.68; PD 68/147	*Solidago floribunda*	Netherlands	GU238182	GU237899	KT389677	GU237687
S. dorenboschii	CBS 426.90; IMI 386093; PD 86/551	*Physostegia virginiana*	Netherlands	GU238185	GU237862	KT389678	GU237690
S. helianthi	CBS 200.87	*Helianthus annuus*	Italy	KT389761	KT389545	KT389683	KT389848
S. heliopsidis	CBS 109182; PD 74/231	*Heliopsis patula*	Netherlands	GU238186	GU237747	KT389679	GU237691
S. hortensis	CBS 104.42	—	Netherlands	GU238198	GU237730	KT389680	GU237703
	CBS 572.85; PD 79/269	*Phaseolus vulgaris*	Netherlands	GU238199	GU237893	KT389681	GU237704
S. inoxydabilis	CBS 425.90; PD 81/520	*Chrysanthemum parthenii*	Netherlands	GU238188	GU237861	KT389682	GU237693
S. loticola	CBS 562.81; PDDCC 6884	*Lotus pedunculatus*	New Zealand	GU238192	GU237890	KT389684	GU237697
S. lupini	CBS 101494; PD 98/5247	*Lupinus albus*	UK	GU238194	GU237724	KT389685	GU237699
S. oculo-hominis	CBS 634.92; IMI 193307	*Human corneal ulcer*	USA	GU238196	GU237901	KT389686	GU237701
S. papillatus	CGMCC 3.18367; LC 8169	*Rumex nepalensis*	China	KY742279	KY742125	KY742191	KY742367
	LC 8170	*Rumex nepalensis*	China	KY742280	KY742126	KY742192	KY742368
	LC 8171	Boraginaceae	China	KY742281	KY742127	KY742193	KY742369

Supplementary table 2. (Continued).

Species	Strain number	Host, substrate	Country	GenBank accession numbers			
				LSU	ITS	rpb2	tub2
S. rudbeckiae	CBS 109180; PD 79/175	*Rudbeckia bicolor*	Netherland	GU23819	GU237745	—	GU23770
S. tanaceti	CBS 131484	*Tanacetum cinerariifolium*	Australia	JQ897461	NR_11172	—	JQ897496
S. trachelii	CBS 379.91; PD 77/675	*Campanula isophylla*	Netherland	GU23817	GU237850	KT38968	GU23767
	CBS 384.68	*Campanula isophylla*	Sweden	GU23817	GU237856	—	GU23767
S. valerianellae	CBS 273.92; PD 82/43	*Valerianella locusta*	Netherland	GU23820	GU237819	—	GU23770
	CBS 329.67; PD 66/302	*Valerianella locusta* var. *oleracea*	Netherland	GU23820	GU237832	—	GU23770
Vacuiphoma bulgarica	CBS 357.84; FMR 14917	*Trachystemon orientale*	Bulgaria	GU23805	GU237837	LT623256	GU23758
V. oculihominis	UTHSC:DI16-308; FMR 13801	*Human superficial tissue*	USA	LN90745	LT592954	LT593093	LT593023
Xenodidymella	CBS 195.36	*Rubus idaeus*	Netherland	KT38976	KT389548	—	KT38985
X. applanata	CBS 205.63	*Rubus idaeus*	Netherland	GU23799	GU237798	KP33040	GU23755
	CBS 115577	*Rubus idaeus*	Sweden	KT38976	KT389546	KT38968	KT38985
	CBS 115578	*Rubus arcticus notho sp.*	Sweden	KT38976	KT389547	—	KT38985
X. asphodeli	CBS 375.62	*Asphodelus albus*	France	KT38976	KT389549	KT38968	—
	CBS 499.72	*Asphodelus ramosus*	Italy	KT38976	KT389550	—	KT38985
X. catariae	CBS 102635; PD 77/1131	*Nepeta cataria*	Netherland	GU23796	GU237727	KP33040	GU23752
X. humicola	CBS 220.85; PD 71/1030	*Franseria* sp.	USA	GU23808	GU237800	KP33042	GU23761

Supplementary Table 3. The GenBank accession numbers of the host plants used for co-evolution analysis.

Host	18S	matK	rbcL
Ageratina adenophora	AY576867.1	MF159398.1	MF135333.1
Ampelopsis delavayana	JF975891.1	JF953241.1	HM223363.1
Celtis tetrandra	JF317361.1	JF317420.1	JF317479.1
Quercus glauca	-	AB060062.1	AB060571.1
Achyranthes bidentata	JQ621855.1	LT992574.1	GQ436715.1
Rubia cordifolia	-	KP190175.1	KP137603.1
Reinwardtia indica	AF207005.1	AB048380.1	KX527428.1
Arthraxon hispidus	DQ006027.1	KF163819.1	KF163492.1
Urena lobata	MH768244.1	EF207260.1	MH767648.1
Fallopia multiflora	EF153716.1	EF159150.1	DQ539661.1
Abelmoschus moschatus	JQ230968.1	MF349855.1	MH588543.1
Zehneria maysorensis	KY523259.1	KY523337.1	-
Pueraria peduncularis	-	-	GQ436362.1
Lindera communis	-	AF244406.1	KX546879.1
Petunia hybrida	-	EF439018.1	KX783965.1

Supplementary Table 4. Information of the fungal isolates used in this study.

Sample ID	City	County	Invasion or not	Host, substrate	Genus	Sample ID	City	County	Invasion area	Host, substrate	Genus
A393	Dali	JC	No	*Cyclobalanopsis glauca*	EPI	A826	Zhaotong	YL	No	*Betula alnoides*	DID
A381	Dali	JC	No	*Alnus nepalensis*	DID	A827	Zhaotong	YL	No	*Betula alnoides*	REM
A382	Dali	JC	No	*Alnus nepalensis*	DID	A713	Zhaotong	ZX	No	*Rubus parvifolius*	EPI
A367	Dali	JC	No	*Cynanchum otophyllum*	NOT	A719	Zhaotong	ZX	No	*Betula alnoides*	EPI
A369	Dali	JC	No	*Cynanchum otophyllum*	BOE	A724	Zhaotong	ZX	No	*Betula alnoides*	DID
A430	Nujiang	LP	No	*Alnus nepalensis*	DID	A606	Zhaotong	ZX	No	*Cynanchum otophyllum*	EPI
A408	Nujiang	LP	No	*Dioscorea hemsleyi*	DID	A609	Zhaotong	ZX	No	*Cynanchum otophyllum*	REM
A410	Nujiang	LP	No	*Dioscorea hemsleyi*	DID	G445	Dali	WS	Yes	Leaf spot of *A. adenophora*	DID
A679	Zhaotong	CB	No	*Cyclobalanopsis glauca*	DID	G446	Dali	WS	Yes	Leaf spot of *A. adenophora*	D1
A660	Zhaotong	CB	No	*Fallopia multiflora*	BOE	G447	Dali	WS	Yes	Leaf spot of *A. adenophora*	D1
A696	Zhaotong	CB	No	*Quercus variabilis*	EPI	G450	Dali	WS	Yes	Leaf spot of *A. adenophora*	D1
A577	Zhaotong	CB	No	*Gonostegia hirta*	DID	G452	Dali	WS	Yes	Leaf spot of *A. adenophora*	D1
A671	Zhaotong	CB	No	*Amygdalus persica*	DID	G472	Dali	WS	Yes	Leaf spot of *A. adenophora*	EPI
A566	Zhaotong	XCB	No	*Betula alnoides*	EPI	G473	Dali	WS	Yes	Leaf spot of *A. adenophora*	EPI
A628	Zhaotong	XCB	No	*Dioscorea hemsleyi*	REM	G478	Dali	WS	Yes	Leaf spot of *A. adenophora*	D1
A804	Zhaotong	YL	No	*Alnus nepalensis*	EPI	G483	Dali	WS	Yes	Leaf spot of *A. adenophora*	D1
A825	Zhaotong	YL	No	*Betula alnoides*	EPI	G485	Dali	WS	Yes	Leaf spot of *A. adenophora*	D1

Supplementary table 4. (Continued).

Sample ID	City	County	Invasion or not	Host, substrate	Genus	Sample ID	City	County	Invasion area	Host, substrate	Genus
G499	Dali	WS	Yes	Leaf spot of *A. adenophora*	DID	K93	Kunming	XS	Yes	Canopy air of *A. adenophora*	EPI
G503	Dali	WS	Yes	Leaf spot of *A. adenophora*	D1	A781	Kunming	XS	Yes	*Eupatorium fortunei*	EPI
G505	Dali	WS	Yes	Leaf spot of *A. adenophora*	D1	A783	Kunming	XS	Yes	*Eupatorium fortunei*	EPI
G516	Dali	WS	Yes	Leaf spot of *A. adenophora*	D1	A780	Kunming	XS	Yes	*Commelina communis*	EPI
G575	Dali	WS	Yes	Leaf spot of *A. adenophora*	EPI	A788	Kunming	XS	Yes	*Eupatorium heterophyllum*	EPI
A42	Dali	NJ	Yes	Canopy air of *A. adenophora*	EPI	A790	Kunming	XS	Yes	*Eupatorium heterophyllum*	EPI
A466	Dali	YL	Yes	*Cyclobalanopsis glauca*	DID	A791	Kunming	XS	Yes	*Eupatorium heterophyllum*	EPI
Y63	Dali	YL	Yes	Root of *A. adenophora*	D1	A796	Kunming	XS	Yes	*Eupatorium heterophyllum*	EPI
Y98	Dali	YL	Yes	Root of *A. adenophora*	D1	A798	Kunming	XS	Yes	*Eupatorium heterophyllum*	EPI
Y188	Dali	YL	Yes	Rotted leaf of *A. adenophora*	D1	K119	Kunming	XS	Yes	Stem of *A. adenophora*	D1
Y122	Dali	YL	Yes	*A. adenophora*	D2	K120	Kunming	XS	Yes	Stem of *A. adenophora*	D1
A732	Kunming	XS	Yes	*Alnus nepalensis*	DID	K195	Kunming	XS	Yes	Stem of *A. adenophora*	D1
A733	Kunming	XS	Yes	*Alnus nepalensis*	NOT	G10	Kunming	XS	Yes	Leaf spot of *A. adenophora*	D2
K103	Kunming	XS	Yes	Canopy air of *A. adenophora*	EPI	G135	Kunming	XS	Yes	Leaf spot of *A. adenophora*	D1
K11	Kunming	XS	Yes	Canopy air of *A. adenophora*	EPI	G14	Kunming	XS	Yes	Leaf spot of *A. adenophora*	D2
K16	Kunming	XS	Yes	Canopy air of *A. adenophora*	EPI	G15	Kunming	XS	Yes	Leaf spot of *A. adenophora*	D1
K257	Kunming	XS	Yes	Canopy air of *A. adenophora*	EPI	G159	Kunming	XS	Yes	Leaf spot of *A. adenophora*	STA
K66	Kunming	XS	Yes	Canopy air of *A. adenophora*	EPI	G168	Kunming	XS	Yes	Leaf spot of *A. adenophora*	D1
K77	Kunming	XS	Yes	Canopy air of *A. adenophora*	EPI	G170	Kunming	XS	Yes	Leaf spot of *A. adenophora*	D2
K81	Kunming	XS	Yes	Canopy air of *A. adenophora*	EPI	G187	Kunming	XS	Yes	Leaf spot of *A. adenophora*	D2

Supplementary table 4. (Continued).

Sample ID	City	County	Invasion or not	Host, substrate	Genus	Sample ID	City	County	Invasion area	Host, substrate	Genus
G188	Kunming	XS	Yes	Leaf spot of *A. adenophora*	D2	G65	Kunming	XS	Yes	Leaf spot of *A. adenophora*	D1
G191	Kunming	XS	Yes	Leaf spot of *A. adenophora*	D2	K148	Kunming	XS	Yes	*A. adenophora*	D1
G196	Kunming	XS	Yes	Leaf spot of *A. adenophora*	D2	K169	Kunming	XS	Yes	*A. adenophora*	D1
G204	Kunming	XS	Yes	Leaf spot of *A. adenophora*	D2	K216	Kunming	XS	Yes	*A. adenophora*	D1
G214	Kunming	XS	Yes	Leaf spot of *A. adenophora*	D2	K222	Kunming	XS	Yes	*A. adenophora*	EPI
G215	Kunming	XS	Yes	Leaf spot of *A. adenophora*	D2	K229	Kunming	XS	Yes	*A. adenophora*	D1
G216	Kunming	XS	Yes	Leaf spot of *A. adenophora*	D2	K51	Kunming	XS	Yes	*A. adenophora*	EPI
G236	Kunming	XS	Yes	Leaf spot of *A. adenophora*	D2	C121	Lincang	CY	Yes	Rhizosphere of *A. adenophora*	REM
G239	Kunming	XS	Yes	Leaf spot of *A. adenophora*	D2	C195	Lincang	CY	Yes	Rhizosphere of *A. adenophora*	ALL
G240	Kunming	XS	Yes	Leaf spot of *A. adenophora*	D1	C74	Lincang	CY	Yes	Canopy air of *A. adenophora*	REM
G243	Kunming	XS	Yes	Leaf spot of *A. adenophora*	STA	C77	Lincang	CY	Yes	Canopy air of *A. adenophora*	STA
G25	Kunming	XS	Yes	Leaf spot of *A. adenophora*	STA	C80	Lincang	CY	Yes	Canopy air of *A. adenophora*	D1
G255	Kunming	XS	Yes	Leaf spot of *A. adenophora*	D1	C153	Lincang	CY	Yes	Root of *A. adenophora*	D1
G267	Kunming	XS	Yes	Leaf spot of *A. adenophora*	D2	C158	Lincang	CY	Yes	Root of *A. adenophora*	D1
G271	Kunming	XS	Yes	Leaf spot of *A. adenophora*	STA	C20	Lincang	CY	Yes	Root of *A. adenophora*	DID
G274	Kunming	XS	Yes	Leaf spot of *A. adenophora*	D2	C65	Lincang	CY	Yes	Root of *A. adenophora*	D1
G283	Kunming	XS	Yes	Leaf spot of *A. adenophora*	DID	G1051	Lincang	CY	Yes	Leaf spot of *A. adenophora*	REM
G4	Kunming	XS	Yes	Leaf spot of *A. adenophora*	D2	G806	Lincang	CY	Yes	Leaf spot of *A. adenophora*	DID
G55	Kunming	XS	Yes	Leaf spot of *A. adenophora*	EPI	G873	Lincang	CY	Yes	Leaf spot of *A. adenophora*	REM
G56	Kunming	XS	Yes	Leaf spot of *A. adenophora*	D2	G876	Lincang	CY	Yes	Leaf spot of *A. adenophora*	D1

Supplementary table 4. (Continued).

Sample ID	City	County	Invasion or not	Host, substrate	Genus	Sample ID	City	County	Invasion area	Host, substrate	Genus
G926	Lincang	CY	Yes	Leaf spot of *A. adenophora*	DID	G405	Lincang	YX	Yes	Leaf spot of *A. adenophora*	D1
G937	Lincang	CY	Yes	Leaf spot of *A. adenophora*	D1	G406	Lincang	YX	Yes	Leaf spot of *A. adenophora*	D1
G940	Lincang	CY	Yes	Leaf spot of *A. adenophora*	D1	A869	Puer	LC	Yes	*Betula alnoides*	REM
C222	Lincang	CY	Yes	Rotted leaf of *A. adenophora*	D1	A872	Puer	LC	Yes	*Betula alnoides*	EPI
C224	Lincang	CY	Yes	Rotted leaf of *A. adenophora*	D1	G1312	Puer	LC	Yes	Leaf spot of *A. adenophora*	REM
C111	Lincang	CY	Yes	*A. adenophora*	EPI	G1338	Puer	LC	Yes	Leaf spot of *A. adenophora*	REM
C113	Lincang	CY	Yes	*A. adenophora*	DID	G1461	Puer	LC	Yes	Leaf spot of *A. adenophora*	D1
C37	Lincang	CY	Yes	*A. adenophora*	EPI	G1646	Puer	LC	Yes	Leaf spot of *A. adenophora*	D1
C38	Lincang	CY	Yes	*A. adenophora*	EPI	N79	Puer	NE	Yes	Canopy air of *A. adenophora*	PAR
C42	Lincang	CY	Yes	*A. adenophora*	EPI	N83	Puer	NE	Yes	Canopy air of *A. adenophora*	EPI
C82	Lincang	CY	Yes	*A. adenophora*	LEP	N9	Puer	NE	Yes	Canopy air of *A. adenophora*	EPI
C83	Lincang	CY	Yes	*A. adenophora*	EPI	G1489	Puer	NE	Yes	Leaf spot of *A. adenophora*	D1
C84	Lincang	CY	Yes	*A. adenophora*	EPI	G1534	Puer	NE	Yes	Leaf spot of *A. adenophora*	REM
C87	Lincang	CY	Yes	*A. adenophora*	EPI	G1548	Puer	NE	Yes	Leaf spot of *A. adenophora*	REM
C88	Lincang	CY	Yes	*A. adenophora*	EPI	N16	Puer	NE	Yes	*A. adenophora*	NEOA
C89	Lincang	CY	Yes	*A. adenophora*	EPI	N27	Puer	NE	Yes	*A. adenophora*	EPI
C96	Lincang	CY	Yes	*A. adenophora*	EPI	N28	Puer	NE	Yes	*A. adenophora*	DID
A1021	Lincang	YX	Yes	*Betula alnoides*	EPI	N64	Puer	NE	Yes	*A. adenophora*	EPI
A1002	Lincang	YX	Yes	*Gonostegia hirta*	EPI	N84	Puer	NE	Yes	*A. adenophora*	DID
A958	Lincang	YX	Yes	*Nicotiana tabacum*	BOE	N86	Puer	NE	Yes	*A. adenophora*	EPI

Supplementary table 4. (Continued).

Sample ID	City	County	Invasion or not	Host, substrate	Genus	Sample ID	City	County	Invasion area	Host, substrate	Genus
S51	Puer	SM	Yes	Canopy air of *A. adenophora*	DID	G603	Puer	XM	Yes	Leaf spot of *A. adenophora*	D1
S188	Puer	SM	Yes	Root of *A. adenophora*	D1	G644	Puer	XM	Yes	Leaf spot of *A. adenophora*	D2
S102	Puer	SM	Yes	*A. adenophora*	D1	G650	Puer	XM	Yes	Leaf spot of *A. adenophora*	D1
S104	Puer	SM	Yes	*A. adenophora*	D1	G745	Puer	XM	Yes	Leaf spot of *A. adenophora*	D1
A886	Puer	XM	Yes	*Alnus nepalensis*	DID	G775	Puer	XM	Yes	Leaf spot of *A. adenophora*	D1
A902	Puer	XM	Yes	*Alnus nepalensis*	EPI	X69	Puer	XM	Yes	*A. adenophora*	DID
A909	Puer	XM	Yes	*Dioscorea hemsleyi*	EPI	X73	Puer	XM	Yes	*A. adenophora*	DID
A911	Puer	XM	Yes	*Dioscorea hemsleyi*	DID	A68	Yuxi	ES	Yes	*Fallopia multiflora*	EPI
A916	Puer	XM	Yes	*Dioscorea hemsleyi*	BOE	A60	Yuxi	ES	Yes	*Dioscorea hemsleyi*	EPI
X170	Puer	XM	Yes	Stem of *A. adenophora*	D1	A65	Yuxi	ES	Yes	*Dioscorea hemsleyi*	EPI
X174	Puer	XM	Yes	Stem of *A. adenophora*	D1	G1693	Yuxi	ES	Yes	Leaf spot of *A. adenophora*	D1
G1075	Puer	XM	Yes	Leaf spot of *A. adenophora*	DID	G1696	Yuxi	ES	Yes	Leaf spot of *A. adenophora*	D1
G1079	Puer	XM	Yes	Leaf spot of *A. adenophora*	D1	G1698	Yuxi	ES	Yes	Leaf spot of *A. adenophora*	D1
G1131	Puer	XM	Yes	Leaf spot of *A. adenophora*	D1	G1704	Yuxi	ES	Yes	Leaf spot of *A. adenophora*	D1
G1138	Puer	XM	Yes	Leaf spot of *A. adenophora*	D1	G1706	Yuxi	ES	Yes	Leaf spot of *A. adenophora*	D1
G1158	Puer	XM	Yes	Leaf spot of *A. adenophora*	D1	G1710	Yuxi	ES	Yes	Leaf spot of *A. adenophora*	D1
G1185	Puer	XM	Yes	Leaf spot of *A. adenophora*	D1	G1711	Yuxi	ES	Yes	Leaf spot of *A. adenophora*	D1
G1188	Puer	XM	Yes	Leaf spot of *A. adenophora*	D1	G1716	Yuxi	ES	Yes	Leaf spot of *A. adenophora*	BOE
G1214	Puer	XM	Yes	Leaf spot of *A. adenophora*	D1	G1727	Yuxi	ES	Yes	Leaf spot of *A. adenophora*	D1
G1285	Puer	XM	Yes	Leaf spot of *A. adenophora*	D1	G1740	Yuxi	ES	Yes	Leaf spot of *A. adenophora*	EPI

Supplementary table 4. (Continued).

Sample ID	City	County	Invasion or not	Host, substrate	Genus	Sample ID	City	County	Invasion area	Host, substrate	Genus
G1753	Yuxi	ES	Yes	Leaf spot of *A. adenophora*	D1	G1816	Yuxi	ES	Yes	Leaf spot of *A. adenophora*	D1
G1783	Yuxi	ES	Yes	Leaf spot of *A. adenophora*	DID	G1819	Yuxi	ES	Yes	Leaf spot of *A. adenophora*	D1
G1786	Yuxi	ES	Yes	Leaf spot of *A. adenophora*	D1	G1828	Yuxi	ES	Yes	Leaf spot of *A. adenophora*	D1
G1791	Yuxi	ES	Yes	Leaf spot of *A. adenophora*	EPI	G1846	Yuxi	ES	Yes	Leaf spot of *A. adenophora*	D1
G1792	Yuxi	ES	Yes	Leaf spot of *A. adenophora*	D1	G1858	Yuxi	ES	Yes	Leaf spot of *A. adenophora*	D1
G1803	Yuxi	ES	Yes	Leaf spot of *A. adenophora*	D1	G1861	Yuxi	ES	Yes	Leaf spot of *A. adenophora*	D1
G1804	Yuxi	ES	Yes	Leaf spot of *A. adenophora*	D1	G1865	Yuxi	ES	Yes	Leaf spot of *A. adenophora*	D1
G1808	Yuxi	ES	Yes	Leaf spot of *A. adenophora*	D1	A89	Yuxi	MJ	Yes	*Camellia sinensis*	EPI
G1813	Yuxi	ES	Yes	Leaf spot of *A. adenophora*	D1	A114	Yuxi	MJ	Yes	*Dioscorea hemsleyi*	EPI
G1814	Yuxi	ES	Yes	Leaf spot of *A. adenophora*	D1						

Supplementary table 5. OTUs defined based on different loci or similarity.

ID	ITS uni	ITS 97%	4 LOCI	GENUS	ID	ITS uni	ITS 97%	4 LOCI	GENUS
C195	OTU35	OTU1	OTU95	ALL	G1792	OTU1	OTU1	OTU3	D1
A660	OTU9	OTU2	OTU111	BOE	G1804	OTU1	OTU1	OTU3	D1
G1716	OTU9	OTU2	OTU35	BOE	G1814	OTU1	OTU1	OTU3	D1
A916	OTU30	OTU2	OTU89	BOE	G255	OTU1	OTU1	OTU3	D1
A958	OTU9	OTU2	OTU90	BOE	G446	OTU1	OTU1	OTU3	D1
A369	OTU9	OTU2	OTU99	BOE	G483	OTU1	OTU1	OTU3	D1
C158	OTU1	OTU1	OTU1	D1	G65	OTU1	OTU1	OTU3	D1
G1079	OTU1	OTU1	OTU1	D1	G775	OTU1	OTU1	OTU3	D1
G1185	OTU1	OTU1	OTU1	D1	K119	OTU1	OTU1	OTU3	D1
G1285	OTU1	OTU1	OTU1	D1	K229	OTU1	OTU1	OTU3	D1
G1489	OTU1	OTU1	OTU1	D1	S188	OTU1	OTU1	OTU3	D1
G1706	OTU1	OTU1	OTU1	D1	G168	OTU1	OTU1	OTU36	D1
G1710	OTU1	OTU1	OTU1	D1	G15	OTU1	OTU1	OTU37	D1
G1808	OTU1	OTU1	OTU1	D1	G1461	OTU1	OTU1	OTU38	D1
G1816	OTU1	OTU1	OTU1	D1	G1711	OTU1	OTU1	OTU4	D1
G1846	OTU1	OTU1	OTU1	D1	G1786	OTU1	OTU1	OTU4	D1
G1858	OTU1	OTU1	OTU1	D1	G1813	OTU1	OTU1	OTU4	D1
G1861	OTU1	OTU1	OTU1	D1	G1865	OTU1	OTU1	OTU4	D1
G405	OTU1	OTU1	OTU1	D1	G240	OTU1	OTU1	OTU4	D1
G406	OTU1	OTU1	OTU1	D1	G450	OTU1	OTU1	OTU4	D1
G447	OTU1	OTU1	OTU1	D1	G603	OTU1	OTU1	OTU4	D1
G503	OTU1	OTU1	OTU1	D1	S104	OTU1	OTU1	OTU4	D1
G505	OTU1	OTU1	OTU1	D1	Y63	OTU1	OTU1	OTU44	D1
G516	OTU1	OTU1	OTU1	D1	X170	OTU1	OTU1	OTU47	D1
G650	OTU1	OTU1	OTU1	D1	S102	OTU1	OTU1	OTU49	D1
G940	OTU1	OTU1	OTU1	D1	G1131	OTU1	OTU1	OTU5	D1
Y188	OTU1	OTU1	OTU1	D1	G1158	OTU1	OTU1	OTU5	D1
C153	OTU1	OTU1	OTU107	D1	G1188	OTU1	OTU1	OTU5	D1
G1828	OTU1	OTU1	OTU11	D1	G1693	OTU1	OTU1	OTU5	D1
K169	OTU1	OTU1	OTU11	D1	G1819	OTU1	OTU1	OTU5	D1
X174	OTU1	OTU1	OTU11	D1	G478	OTU1	OTU1	OTU5	D1
C65	OTU1	OTU1	OTU123	D1	G876	OTU1	OTU1	OTU5	D1
C224	OTU1	OTU1	OTU125	D1	G135	OTU1	OTU1	OTU6	D1
C222	OTU1	OTU1	OTU130	D1	G1646	OTU1	OTU1	OTU6	D1
G745	OTU1	OTU1	OTU20	D1	G1698	OTU1	OTU1	OTU6	D1
G452	OTU1	OTU1	OTU22	D1	G1704	OTU1	OTU1	OTU6	D1
G1803	OTU1	OTU1	OTU25	D1	G485	OTU1	OTU1	OTU6	D1
G1138	OTU1	OTU1	OTU3	D1	Y98	OTU1	OTU1	OTU6	D1
G1727	OTU1	OTU1	OTU3	D1	K216	OTU1	OTU1	OTU65	D1
G1753	OTU1	OTU1	OTU3	D1	K195	OTU1	OTU1	OTU66	D1

Supplementary table 5. (Continued).

ID	ITS uni	ITS 97%	4 LOCI	GENUS	ID	ITS uni	ITS 97%	4 LOCI	GENUS
K148	OTU1	OTU1	OTU68	D1	A410	OTU41	OTU2	OTU103	DID
K120	OTU1	OTU1	OTU69	D1	A466	OTU6	OTU2	OTU105	DID
G937	OTU19	OTU1	OTU72	D1	A577	OTU20	OTU2	OTU106	DID
C80	OTU1	OTU1	OTU9	D1	A671	OTU45	OTU2	OTU112	DID
G1214	OTU1	OTU1	OTU9	D1	A679	OTU20	OTU2	OTU113	DID
G1696	OTU1	OTU1	OTU9	D1	C20	OTU16	OTU2	OTU129	DID
G10	OTU2	OTU1	OTU2	D2	A430	OTU6	OTU2	OTU16	DID
G187	OTU2	OTU1	OTU2	D2	A826	OTU6	OTU2	OTU16	DID
G188	OTU2	OTU1	OTU2	D2	G1075	OTU10	OTU2	OTU17	DID
G191	OTU2	OTU1	OTU2	D2	X73	OTU10	OTU2	OTU17	DID
G196	OTU2	OTU1	OTU2	D2	G499	OTU6	OTU2	OTU21	DID
G204	OTU2	OTU1	OTU2	D2	G806	OTU37	OTU2	OTU26	DID
G214	OTU2	OTU1	OTU2	D2	G445	OTU10	OTU2	OTU28	DID
G215	OTU2	OTU1	OTU2	D2	G283	OTU24	OTU2	OTU29	DID
G216	OTU2	OTU1	OTU2	D2	G1783	OTU26	OTU2	OTU33	DID
G239	OTU2	OTU1	OTU2	D2	X69	OTU6	OTU2	OTU46	DID
G267	OTU2	OTU1	OTU2	D2	S51	OTU42	OTU2	OTU48	DID
G274	OTU2	OTU1	OTU2	D2	N84	OTU44	OTU2	OTU52	DID
G4	OTU2	OTU1	OTU2	D2	N28	OTU48	OTU2	OTU56	DID
G644	OTU2	OTU1	OTU2	D2	G926	OTU36	OTU2	OTU58	DID
G14	OTU2	OTU1	OTU39	D2	A732	OTU49	OTU2	OTU74	DID
Y122	OTU2	OTU1	OTU45	D2	A886	OTU10	OTU2	OTU84	DID
G170	OTU2	OTU1	OTU7	D2	A724	OTU47	OTU2	OTU85	DID
G236	OTU2	OTU1	OTU7	D2	A911	OTU16	OTU2	OTU88	DID
G56	OTU2	OTU1	OTU7	D2	C113	OTU18	OTU2	OTU92	DID
A609	OTU8	OTU2	OTU109	REM	A393	OTU3	OTU2	OTU10	EPI
C74	OTU8	OTU2	OTU124	REM	A783	OTU3	OTU2	OTU10	EPI
G1534	OTU5	OTU2	OTU14	REM	A804	OTU3	OTU2	OTU10	EPI
G1548	OTU5	OTU2	OTU14	REM	A42	OTU15	OTU3	OTU104	EPI
A628	OTU8	OTU2	OTU19	REM	A606	OTU3	OTU2	OTU108	EPI
G1051	OTU8	OTU2	OTU19	REM	A65	OTU21	OTU3	OTU110	EPI
G873	OTU5	OTU2	OTU27	REM	A68	OTU21	OTU3	OTU114	EPI
G1338	OTU5	OTU2	OTU40	REM	A696	OTU12	OTU2	OTU115	EPI
G1312	OTU5	OTU2	OTU41	REM	A713	OTU3	OTU2	OTU116	EPI
A827	OTU8	OTU2	OTU81	REM	A719	OTU12	OTU2	OTU117	EPI
A869	OTU5	OTU2	OTU82	REM	C88	OTU13	OTU3	OTU118	EPI
C121	OTU33	OTU2	OTU93	REM	C84	OTU29	OTU3	OTU119	EPI
A381	OTU40	OTU2	OTU100	DID	G472	OTU23	OTU3	OTU12	EPI
A382	OTU6	OTU2	OTU101	DID	G473	OTU23	OTU3	OTU12	EPI
A408	OTU16	OTU2	OTU102	DID	C38	OTU13	OTU3	OTU120	EPI

Supplementary table 5. (Continued).

ID	ITS uni	ITS 97%	4 LOCI	GENUS	ID	ITS uni	ITS 97%	4 LOCI	GENUS
C87	OTU27	OTU3	OTU121	EPI	K16	OTU28	OTU1	OTU67	EPI
C37	OTU4	OTU3	OTU122	EPI	A1002	OTU4	OTU3	OTU70	EPI
C83	OTU13	OTU3	OTU126	EPI	K103	OTU18	OTU2	OTU71	EPI
C42	OTU3	OTU2	OTU128	EPI	A89	OTU3	OTU2	OTU73	EPI
A796	OTU3	OTU2	OTU13	EPI	A780	OTU51	OTU3	OTU76	EPI
K77	OTU3	OTU2	OTU13	EPI	A781	OTU11	OTU3	OTU77	EPI
A566	OTU3	OTU2	OTU15	EPI	A790	OTU3	OTU2	OTU78	EPI
A791	OTU3	OTU2	OTU15	EPI	A798	OTU14	OTU2	OTU79	EPI
A114	OTU11	OTU3	OTU18	EPI	A825	OTU7	OTU2	OTU80	EPI
A788	OTU11	OTU3	OTU18	EPI	A872	OTU12	OTU2	OTU83	EPI
G55	OTU43	OTU2	OTU23	EPI	A902	OTU22	OTU3	OTU86	EPI
G575	OTU4	OTU3	OTU24	EPI	A909	OTU22	OTU3	OTU87	EPI
K11	OTU14	OTU2	OTU31	EPI	C111	OTU4	OTU3	OTU91	EPI
G1791	OTU7	OTU2	OTU32	EPI	C89	OTU4	OTU3	OTU94	EPI
G1740	OTU7	OTU2	OTU34	EPI	A60	OTU4	OTU3	OTU96	EPI
C96	OTU4	OTU3	OTU42	EPI	A1021	OTU7	OTU2	OTU97	EPI
N9	OTU14	OTU2	OTU50	EPI	C82	OTU31	OTU5	OTU131	LEP
N86	OTU4	OTU3	OTU51	EPI	N16	OTU32	OTU6	OTU43	NEOA
N83	OTU7	OTU2	OTU53	EPI	A733	OTU50	OTU2	OTU75	NOT
N64	OTU46	OTU2	OTU55	EPI	A367	OTU39	OTU2	OTU98	NOT
N27	OTU7	OTU2	OTU57	EPI	N79	OTU38	OTU4	OTU54	PAR
K93	OTU15	OTU3	OTU59	EPI	C77	OTU34	OTU1	OTU127	STA
K81	OTU52	OTU3	OTU60	EPI	G25	OTU19	OTU1	OTU30	STA
K66	OTU4	OTU3	OTU61	EPI	G159	OTU17	OTU1	OTU8	STA
K51	OTU15	OTU3	OTU62	EPI	G243	OTU17	OTU1	OTU8	STA
K257	OTU3	OTU2	OTU63	EPI	G271	OTU17	OTU1	OTU8	STA
K222	OTU25	OTU2	OTU64	EPI					
